# Suprachiasmatic VIP neurons are required for normal circadian rhythmicity and comprised of molecularly distinct subpopulations

**DOI:** 10.1038/s41467-020-17197-2

**Published:** 2020-09-02

**Authors:** William D. Todd, Anne Venner, Christelle Anaclet, Rebecca Y. Broadhurst, Roberto De Luca, Sathyajit S. Bandaru, Lindsay Issokson, Lauren M. Hablitz, Olga Cravetchi, Elda Arrigoni, John N. Campbell, Charles N. Allen, David P. Olson, Patrick M. Fuller

**Affiliations:** 1 Department of Zoology and Physiology, Program in Neuroscience, University of Wyoming, Laramie, WY 82071 USA; 2 Department of Neurology, Beth Israel Deaconess Medical Center, Division of Sleep Medicine, Harvard Medical School, Boston, MA 02215 USA; 3 Department of Neurobiology, University of Massachusetts Medical School, Worcester, MA 01605 USA; 4 Oregon Institute for Occupational Health Sciences and Department of Behavioral Neuroscience, Oregon Health & Science University, Portland, OR 97239 USA; 5 Department of Biology, University of Virginia, Charlottesville, VA USA; 6 Department of Pediatrics, University of Michigan, Ann Arbor, MI USA

**Keywords:** Circadian mechanisms, Circadian regulation

## Abstract

The hypothalamic suprachiasmatic (SCN) clock contains several neurochemically defined cell groups that contribute to the genesis of circadian rhythms. Using cell-specific and genetically targeted approaches we have confirmed an indispensable role for vasoactive intestinal polypeptide-expressing SCN (SCN^VIP^) neurons, including their molecular clock, in generating the mammalian locomotor activity (LMA) circadian rhythm. Optogenetic-assisted circuit mapping revealed functional, di-synaptic connectivity between SCN^VIP^ neurons and dorsomedial hypothalamic neurons, providing a circuit substrate by which SCN^VIP^ neurons may regulate LMA rhythms. In vivo photometry revealed that while SCN^VIP^ neurons are acutely responsive to light, their activity is otherwise behavioral state invariant. Single-nuclei RNA-sequencing revealed that SCN^VIP^ neurons comprise two transcriptionally distinct subtypes, including putative pacemaker and non-pacemaker populations. Altogether, our work establishes necessity of SCN^VIP^ neurons for the LMA circadian rhythm, elucidates organization of circadian outflow from and modulatory input to SCN^VIP^ cells, and demonstrates a subpopulation-level molecular heterogeneity that suggests distinct functions for specific SCN^VIP^ subtypes.

## Abstract (web summary)

Cell groups in the hypothalamic suprachiasmatic clock contribute to the genesis of circadian rhythms. The authors identified two populations of vasoactive intestinal polypeptide-expressing neurons in the suprachiasmatic nucleus which regulate locomotor circadian rhythm in mice.

## Introduction

Current understanding holds that circadian rhythms are generated within individual cells of the suprachiasmatic nucleus (SCN) and that cell–cell interactions within the SCN network are required to sustain them^1^. One of the emergent properties of these cell–cell circuit interactions is the circadian period, a fundamental property of the SCN circadian clock^2^. While the SCN contains a variety of neuropeptides^3,4^, as well as the fast transmitter GABA^5^, that might variably contribute to SCN network function and hence the ensemble period, previous work has suggested an especially important role for SCN^VIP^ cells as “master pacemakers” of circadian rhythms^6^. In general support of this view, developmental disruption of vasoactive intestinal polypeptide (*VIP*^*−/−*^ mice) or VIP signaling through its cognate receptor, VPAC2 (*Vipr2*^*−/−*^ mice), severely compromises both synchrony among SCN neurons and behavioral rhythms^6–8^. However, more recent studies have suggested an alternate view; that is, a combination of neurons that express arginine vasopressin (AVP)^9^, the dopamine 1a receptor (Drd1a)^10^, or non-VIP neuromedin S (NMS)^11^ within the SCN, are hierarchically dominant to SCN^VIP^ neurons with respect to circadian pacemaking^1^. This hypothesis is surprising given that: (1) despite alterations in circadian period, circadian rhythms are preserved following disruption of the molecular clock in SCN^AVP^ cells^9^, (2) the effects of selective disruption of the molecular clock in SCN^VIP^ neurons have not been determined, and (3) the effect of selective, non-germline ablation of SCN^VIP^ neurons has also not been tested.

Therefore, to more clearly and definitively delineate the role of SCN^VIP^ neurons in the regulation of circadian rhythms, in particular the question of necessity, here we selectively disrupted the molecular clock in SCN^VIP^ cells in mice and recorded circadian rhythms of locomotor activity (LMA), body temperature (Tb), sleep-wake, and wheel running activity. We also used a genetically targeted ablation approach to selectively eliminate SCN^VIP^ cells in adult mice and compared this with genetically targeted lesions of SCN^AVP^ neurons and SCN GABA-ergic neurons, which enabled us to evaluate the effect of non-germline disruption of SCN^VIP^ neurons on circadian rhythms. On the basis of the outcomes of these physiological experiments, which identified necessity of SCN^VIP^ neurons in the regulation of the LMA circadian rhythm, we then extended this experimental work to include a comprehensive interrogation of the cellular and synaptic properties of SCN^VIP^ neurons. To do so, we first employed fiber photometry to evaluate the state-dependent activity dynamics of SCN^VIP^ neurons in vivo. We then used channelrhodopsin (ChR2)-assisted circuit mapping (CRACM) to inform a more detailed understanding of the synaptic output pathways by which SCN^VIP^ cells may produce organismal-level rhythmicity. We similarly employed modified rabies tracing to define synaptic inputs that might modulate SCN^VIP^ activity in vivo. Finally, we used high-throughput single-nuclei sequencing (sNuc-seq) of SCN^VIP^ cells to determine whether SCN^VIP^ neurons comprise distinct subtypes and to define their molecular identities.

## Results

### Selective disruption of the molecular clock in SCN^VIP^ cells

**Fig. 1:**
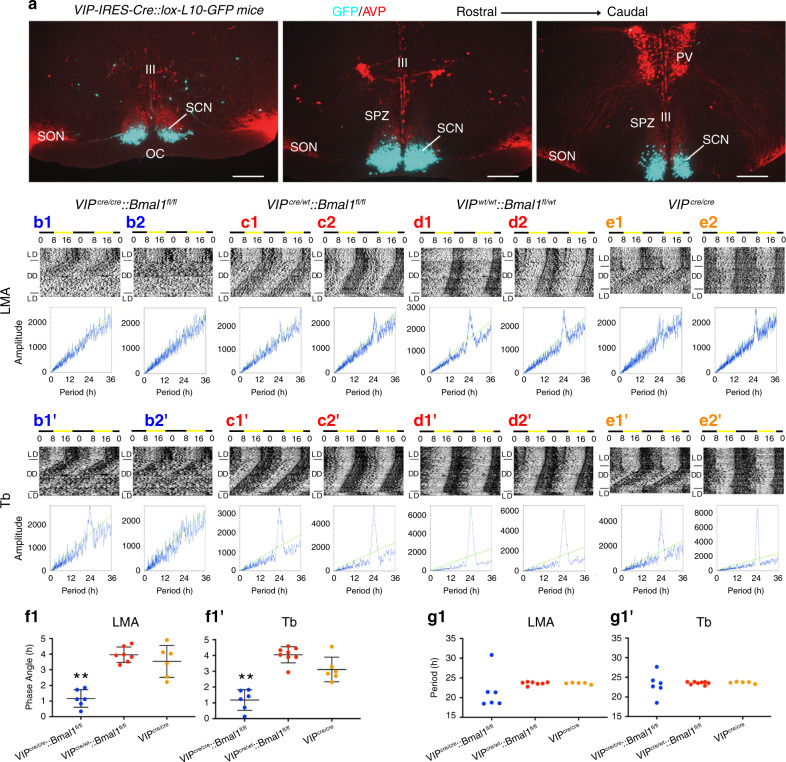
Disruption of the molecular clock in SCN^VIP^ neurons results in eventual loss of the LMA circadian rhythm. **a** Representative microphotographs at three rostro-caudal levels of the ventrolateral SCN from a *VIP-IRES-Cre::lox-L10-GFP* reporter mouse reveals eutopic expression of GFP (scale bar, 300 μm; *n* = 2). See also Fig. S1. **b**-**e** Representative actograms showing locomotor activity (LMA) and body temperature (Tb) circadian rhythms (gray scale: darker represents higher temperature) and associated periodograms during at least 10 days in light/dark (LD), followed by at least three weeks in constant dark (DD), followed by at least 7 days of LD from two *VIP*^*cre/cre*^*::Bmal1*^*fl/fl*^ mice (b1-2, b1’-2’), two *VIP*^*cre/wt*^*::Bmal1*^*fl/fl*^ mice (c1-2, c1’-c2’), two *VIP*^*wt/wt*^*::Bmal1*^*fl/wt*^ mice (d1-2, d1’-d2’) and two *VIP*^*cre/cre*^ mice (e1-2, e1’-2’). Note a profound disruption of LMA rhythms in *VIP*^*cre/cre*^*::Bmal1*^*fl/fl*^ mice (b1-2), whereas these rhythms remain unaltered in *VIP*^*cre/wt*^*::Bmal1*^*fl/fl*^*, VIP*^*wt/wt*^*::Bmal1*^*fl/wt*^, and *VIP*^*cre/cre*^ mice (c1-e2). See also Figure S2. Phase angle of entrainment for LMA (f1) and Tb (f1’) in LD across all mice. Period length of LMA (g1) and Tb (g1’) in DD across all mice. Note that *VIP*^*cre/cre*^*::Bmal1*^*fl/fl*^ mice (*n* = 6) display significantly advanced phase angles of entrainment in LD for both LMA and Tb rhythms compared to *VIP*^*cre/wt*^*::Bmal1*^*fl/fl*^ and *VIP*^*wt/wt*^*::Bmal1*^*fl/wt*^ mice (*n* = 8), and *VIP*^*cre/cre*^ mice (*n* = 5). (For LMA: 1-way ANOVA, *F*_(2,16)_ = 27.57, *P* < 0.0001, Sidak’s post hoc: **P < 0.0001 and *P* < 0.0001; For Tb: 1-way ANOVA, *F*_(2,17)_ = 32.48, *P* < 0.0001, Sidak’s post hoc: **P < 0.0001 and *P* = 0.0002). In DD, *VIP*^*cre/cre*^*::Bmal1*^*fl/fl*^ mice display period lengths outside of the circadian range for LMA rhythms compared to *VIP*^*cre/wt*^*::Bmal1*^*fl/fl*^*, VIP*^*wt/wt*^*::Bmal1*^*fl/wt*^, and *VIP*^*cre/cre*^ mice (note *VIP*^*cre/wt*^*::Bmal1*^*fl/fl*^ and *VIP*^*wt/wt*^*::Bmal1*^*fl/wt*^ mice are included together as *VIP*^*cre/wt*^*::Bmal1*^*fl/fl*^ in f–g). Means ± s.e.m. III, third ventricle; OC, optic chiasm; PV, paraventricular nucleus of the hypothalamus; SPZ, subparaventricular zone; SON, supraoptic nucleus. See also Figs. S3 and S4.

Expression of the *Bmal1* transcription factor within the cellular SCN is both necessary and sufficient for coherent circadian rhythmicity^12,13^. Whether or not a functional cellular clock in SCN^VIP^ neurons, which comprise ~10% of the cellular SCN population^4^, is necessary for establishing and maintaining circadian rhythms remains unclear. To explore this question, we first generated *VIP-IRES-Cre* mice using previously described recombineering techniques^14^ (see Methods). As shown in Fig. 1a, our reporter cross (*VIP-IRES-Cre::R26-loxSTOPlox-L10-GFP*) indicated that expression of the Cre transgene in our mice was restricted to the ventrolateral (core) SCN. To generate mice lacking a functional cellular clock in SCN^VIP^ neurons, we crossed our *VIP-IRES-Cre* mice with mice harboring floxed *Bmal1* alleles (*Bmal1*^*fl/fl*^)^15^. While mice homozygous for the VIP-Cre allele showed the expected complete deletion of *Bmal1* in SCN^VIP^ neurons, mice that were heterozygous for the VIP-Cre allele continued to, unexpectedly, express *Bmal1* in ~70% of VIP neurons (Figure S1). We therefore measured the diurnal and circadian rhythms of LMA and Tb in mice with: (1) complete deletion of *Bmal1* in SCN^VIP^ neurons, and hence loss of the molecular clock in these neurons (*VIP*^*cre/cre*^*::Bmal1*^*fl/fl*^); (2) partial (*VIP*^*cre/wt*^*::Bmal1*^*fl/fl*^ and *VIP*^*wt/wt*^*::Bmal1*^*fl/wt*^) and (3) no deletion of *Bmal1* in SCN^VIP^ cells (*VIP*^*cre/cre*^) (Fig. 1B1-E2’). Under a 12:12 light-dark (LD) cycle, *Bmal1*-deleted mice exhibited a consistent and significantly advanced phase angle of entrainment (~3 h advance) in LMA and Tb (Fig. 1b) as compared to mice with partial (Fig. 1C1-D2’) or no deletion (Fig. 1e) of *Bmal1* in VIP neurons, indicating altered synchronization to the external LD cycle. When the *VIP*^*cre/cre*^*::Bmal1*^*fl/fl*^ mice were released into constant darkness (DD), their LMA rhythms slowly, but also inexorably, became arrhythmic (Fig. 1 B1 and 1B2; 1G1), demonstrating that the molecular clock in SCN^VIP^ cells is necessary for sustaining coherent circadian rhythms of LMA. Despite clear visible effects on the amplitude and architecture of the Tb waveform in DD (Fig. S2), our periodogram analysis revealed that the Tb rhythm (Fig. 1B1’−2’), unlike the LMA rhythm (Fig. 1B1-2), retained a circadian harmonic in the *Bmal1*-deleted mice, with only two of the seven mice exhibiting a period outside of the circadian range (18.5 and 27.7 h, Fig. 1G1’). We finally noted that non-crossed *VIP*^*cre/cre*^ mice (i.e., parent strain in homozygous condition) under 12-12 LD conditions displayed a more variable, although not statistically advanced or delayed, phase angle of entrainment in LMA and Tb (Fig. 1F1-1’) as compared with mice heterozygous for the cre allele or wild-type littermate controls. *VIP*^*cre/cre*^ mice also displayed a range of free running Tb and LMA periods in DD (Fig. 1E1-2, 1E1’−2’; 1G1-1’) that were comparable to those expressed by *VIP*^*cre/wt*^*::Bmal1*^*fl/fl*^ and *VIP*^*wt/wt*^*::Bmal1*^*fl/wt*^ mice. We interpret the more variable phase angle of entrainment as evidence of a possible hypomorphic allele in the *VIP*^*cre/cre*^ condition, although we did not observe a reduction in *VIP* mRNA, and hence putative production of VIP, in *VIP*^*cre/cre*^ homozygous state as compared with mice heterozygous for the cre allele or wild-type littermate controls (Fig. S3). As a genetic comparator, we deleted Bmal1 in AVP neurons by crossing *AVP-ires-Cre* mice^14^ with *Bmal1*^*fl/fl*^ mice (*AVP*^*cre/cre*^*::Bmal*^*fl/fl*^) and analyzed their LMA and Tb rhythms under LD and DD (Fig. S4). We again found that complete deletion of Bmal1 required that the *AVP-ires-Cre* mice be in the homozygous state (i.e., *AVP*^*cre/cre*^); unfortunately, this also produced profound diabetes insipidus in these mice, likely secondary to disruption of AVP function (allele itself or *Bmal1*) in the supraoptic hypothalamus. Regardless, we found that deletion of *Bmal1* from AVP neurons did not produce arrhythmicity in LMA or Tb under DD conditions, but did result in a lengthening of the circadian period of both rhythms (Fig. S4) in the *AVP*^*cre/cre*^*::Bmal*^*fl/fl*^ mice, a finding that recapitulates that previously reported by Mieda and colleagues^9^. Taken together, these findings clearly demonstrate that a functional molecular clock in SCN^VIP^, but not SCN^AVP^, neurons is necessary for circadian rhythms of LMA, and that the absence of this clock in SCN^VIP^ neurons also affects the expression, including phasing and amplitude, of the circadian rhythm of Tb.

### SCN^VIP^ neurons and the circadian rhythm of sleep-wake

**Fig. 2:**
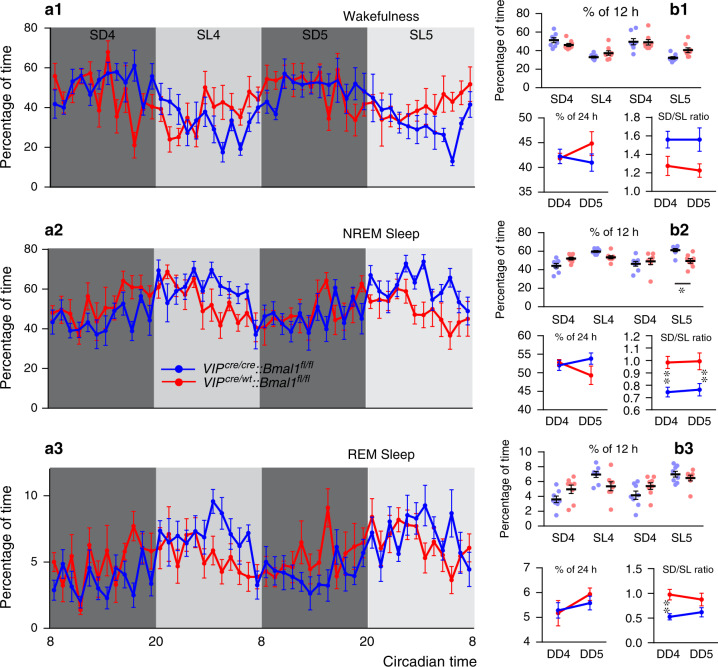
Disruption of the molecular clock in SCN^VIP^ neurons reduces the amplitude of the sleep-wake circadian rhythm. **a** Hourly amounts (mean ± s.e.m.) of wakefulness (a1), NREM sleep (a2) and REM sleep (a3) in *VIP*^*cre/cre*^*::Bmal1*^*fl/fl*^ (blue, *n* = 8) and *VIP*^*cre/wt*^*::Bmal1*^*fl/fl*^ (red, *n* = 8) mice during the 4th and 5th day in constant darkness. **b** Upper panels: Amounts (mean ± s.e.m.) of the vigilance stages during the 4th and 5th subjective dark (SD4, SD5) and subjective light (SL4, SL5) periods. Lower panels: 24 h amounts (mean ± s.e.m.) of the vigilance stages during the 4th and 5th day in constant darkness (DD4, DD5; lower left panels) and subjective dark to subjective light (D/L) ratio for each vigilance stage during the 4th and 5th day in constant darkness (lower right panels). *n* = 8 mice per genotype,*p < 0.05, **p < 0.01, two-way ANOVA followed by a post hoc Bonferroni test. See also Figs. S5 and S6. Note: *VIP*^*cre/wt*^*::Bmal1*^*fl/fl*^ *and VIP*^*wt/wt*^*::Bmal1*^*fl/wt*^ mice are included together under *VIP*^*cre/wt*^*::Bmal1*^*fl/fl*^ in all panels.

The SCN plays an important role in regulating the timing and architecture of the sleep-wake cycle^16,17^. Additional data suggest a possible role for the SCN in regulating the amount of sleep and wake, although this remains a point of controversy^18,19^. A decrease in rapid-eye movement (REM) sleep has also been reported in VIP^−/−^ mice^20^, although whether or not these effects on REM sleep in the VIP^−/−^ mice link to SCN^VIP^ or extra-SCN^VIP^ regulation of REM sleep remains unclear. Regardless, the cellular and synaptic bases by which the SCN clock, including possibly SCN^VIP^ neurons, might regulate the sleep-wake cycle remains to be determined. Given our foregoing findings, we asked whether SCN^VIP^ neurons, or the molecular clock thereof, might contribute to the regulation of the sleep-wake cycle. We did so by recording the electroencephalogram (EEG) to monitor wake, REM and non-REM (NREM) sleep, in the same cohorts of mice as used for the Tb and LMA recordings, while mice were under DD. We found that NREM sleep was increased at the beginning of the subjective day in homozygous *Bmal1*-deleted mice as compared to mice with partial *Bmal1* deletions. And while we did not observe an effect of genotype on the quantity of each vigilance state, as measured over the 24 h period (Fig. 2a), we did find that *Bmal1*-deleted mice exhibited decreased NREM sleep during the subjective light phase, leading to an apparent redistribution of sleep-wake activity (as demonstrated by a subjective dark to subjective light ratio (SD/SL) that is closer to 1 (Fig. 2B2), suggesting a potential role for SCN^VIP^ neurons in regulating the amplitude of the sleep-wake cycle. In order to test if disruption of the molecular clock in SCN^VIP^ neurons affected sleep quality, we examined the vigilance state episodes during the SD and SL periods, as well as performed an analysis of the cortical EEG spectral power. Aside from sporadic individual differences in wakefulness episode duration, *Bmal1*-deleted mice did not demonstrate differences in sleep fragmentation or consolidation as compared to mice with partial *Bmal1* deletions (Fig. S5). Similarly, cortical EEG power distribution across the 24 h day revealed only a few significant differences, e.g., decreased delta power during NREM sleep at one isolated time point, in frequency bands between groups (Fig. S6).

### Selective ablation of SCN^VIP^ cells

**Fig. 3:**
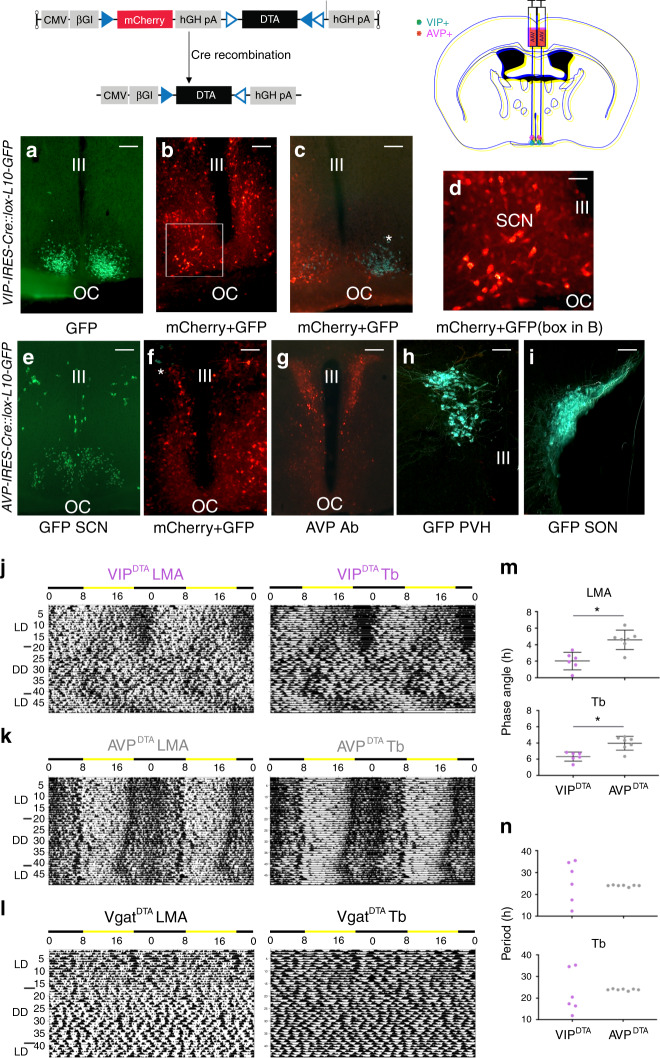
Cell-type specific ablations of SCN^VIP^, but not SCN^AVP^, neurons result in loss of LMA circadian rhythm. (Top) DTA vector construct containing non-Cre-dependent mCherry and Cre-dependent DTA sequences and schematic of AAV-DTA SCN injections in *VIP-IRES-Cre*, *AVP-IRES-Cre* and *Vgat-IRESCre* mice. **a** GFP-expressing VIP neurons in the ventral SCN of a *VIP-IRES-Cre::lox-L10-GFP* mouse (representative example from *n* = 3 mice; scale bar, 150 μm). **b** Representative photomicrograph from a *VIP-IRES-Cre::lox-L10-GFP* mouse that received bilateral AAV-DTA injections. Note that GFP-expressing SCN^VIP^ neurons are absent but that surviving non-VIP neurons express mCherry (scale bar, 150 μm; *n* = 6). **c** Representative photomicrograph from a *VIP-IRES-Cre::lox-L10-GFP* mouse that received a unilateral AAV-DTA injection. An asterisk marks surviving GFP-expressing SCN^VIP^ neurons (cyan) on the non-injected side whereas mCherry-labeled neurons (red) and the absence of GFP-expressing SCN^VIP^ neurons on the contralateral side, indicate complete unilateral ablation (scale bar, 150 μm; *n* = 6). **d** Higher magnification of dual-color box in (b), definitively confirming the absence of GFP (cyan) following near total ablation of SCN^VIP^ neurons (scale bar, 50 μm). **e** GFP-expressing AVP neurons in the dorsal SCN of a AVP-IRES-Cre::lox-L10- GFP mouse (scale bar, 150 μm; representative example from *n* = 6 mice). **f** Representative photomicrograph from a *AVP-IRES-Cre::lox-L10-GFP* mouse that received bilateral AAV-DTA injections. Asterisk marks surviving GFP-labeled (cyan) AVP cells (scale bar, 150 μm; *n* = 6). **g**, **h** Representative cases showing intact immunolabelled (g) and L10-GFP (h) AVP neurons in the paraventricular nucleus (PVH) of two separate mice *AVP-IRES-Cre::lox-L10-GFP* that received bilateral AAV-DTA SCN injections (scale bar, 150 μm; *n* = 6). **i** Intact AVP neurons in supraoptic nucleus (SON), same mouse as H (scale bar, 150 μm; *n* = 6). **j**, **k** Actogram (LMA, locomotor activity) and body temperature (Tb) (gray scale: darker represents higher temperature) during at least 7 days in light/dark (LD), followed by at least 3 weeks in constant dark (DD), followed by at least 7 days of LD. **j** Bilateral ablation of SCN^VIP^ neurons in a *VIP-IRES-Cre* mouse disrupts LMA and Tb circadian rhythms. **k** Bilateral ablation of SCN^AVP^ neurons in an *AVP-IRES-Cre* mouse leaves LMA and Tb rhythms intact. **l** Bilateral ablation of SCN^Vgat^ neurons in a *Vgat-IRES-Cre* mouse abolishes all rhythmicity (*n* = 3). **m** In LD, ablation of SCN^VIP^ neurons results in advanced phase angles of entrainment. Lines represent mean ± s.e.m. (*n* = 6 VIP^DTA^ mice, *n* = 7 AVP^DTA^ mice; two-tailed unpaired *t*-tests: LMA, *t*_(11)_ = 4.104, *p = 0.0017; Tb, *t*_(11)_ = 4.036: *p = 0.002). **n** In DD, ablation of SCN^VIP^ neurons results in periods outside circadian range, whereas ablation of SCN^AVP^ neurons maintains stable periodicity. Abbreviations: III, third ventricle; OC, optic chiasm.

Nearly all in vivo work to date on SCN^VIP^ neurons, including the foregoing experiments in this study, have been performed in developmental mutant models, which have included transgenic crosses. While these models have provided important insights into the neurobiology of the SCN pacemaker, they also have important limitations, namely that developmental disruption of transcription factors, receptors, and neuropeptides, can complicate interpretation secondary to unintended or otherwise unrecognized effects on intercellular signaling and overall network function^10^. We therefore pursued a genetically targeted approach for chronically disrupting SCN^VIP^ neuron function in adult mice. To do this, we placed bilateral injections of a viral vector expressing a Cre-dependent diptheria toxin (DIO-DTA-AAV) into the SCN of *VIP-IRES-Cre* mice (Fig. 3). We also placed bilateral injections of this same toxin into the SCN of *AVP-ires-Cre* mice and *Vgat-ires-Cre* mice to serve as genetic comparators. While the DIO-DTA-AAV has been previously validated^21,22^, and exhibits exquisite selectivity in producing cell death, we confirmed histologically that bilateral injections of this vector into the SCN of *VIP-ires-Cre* (VIP^DTA^) and *AVP-ires-Cre* (AVP^DTA^) mice resulted in cell-specific ablation, with no indication of “off target” cell death (Fig. 3a–i). As shown in Fig. 3j–n, VIP^DTA^ mice with complete lesions (*n* = 6; >95% of VIP cell loss bilaterally) demonstrated a phase angle of entrainment of LMA and Tb in LD that was significantly advanced as compared with AVP^DTA^ mice, and more strikingly, both the Tb and LMA rhythms of these mice became severely fragmented in DD, with all mice exhibiting periods outside of the circadian range (12.33–30.5 h, shortest and longest tau; also see DD Tb waveform in Fig S3). By comparison, AVP^DTA^ mice with complete lesions (*n* = 6) exhibited high-amplitude rhythms of LMA and Tb in DD. Of note, AVP^DTA^ mice did not show the diabetic insipidus phenotype observed in the *AVP*^*cre/cre*^*::Bmal*^*fl/fl*^ mice, reinforcing the extra-SCN etiological basis of this phenotype. Finally, bilateral injections of DIO-DTA-AAV into the SCN of *Vgat-IRES-Cre* mice approximated a near complete cellular lesion of the SCN (due to the near ubiquitous expression of *Vgat* in the cellular SCN^23^) and produced a more dramatic phenotype than seen in the VIP^DTA^ mice, including complete arrhythmicity of LMA and Tb in LD as well as in DD (Fig. 3l). The apparent absence of light-masking by the LD cycle in the *Vgat*^DTA^ mice may reflect the intensity of the light in our LD cycle, but may also link to some DTA-induced cell loss in the dorsally adjacent subparaventricular zone (SPZ), which is largely GABAergic (*Vgat*+) and is a region previously shown to play a role in LD masking^24,25^.

### Selective ablation of SCN^VIP^ cells and sleep-wake

**Fig. 4:**
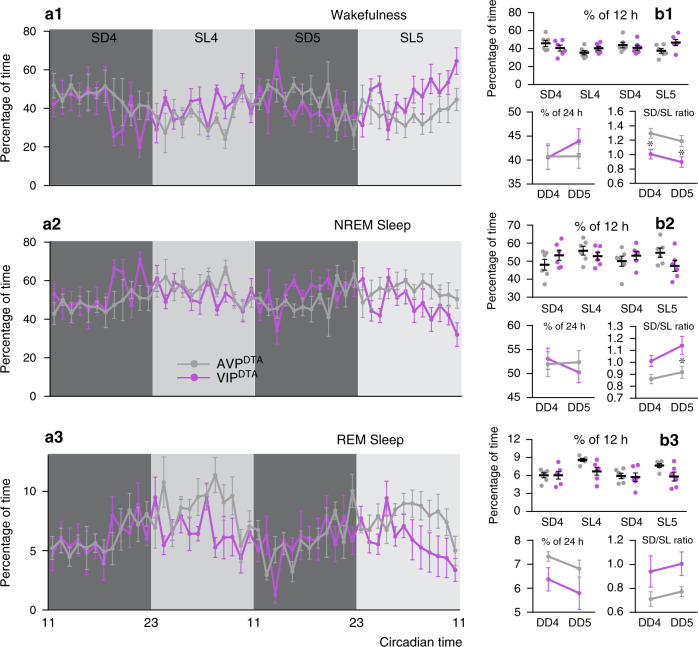
Cell-type specific ablation of SCN^VIP^ neurons, but not SCN^AVP^ neurons, reduces the amplitude of the sleep-wake circadian rhythm. **a** Hourly amounts (mean ± s.e.m.) of wakefulness (a1), NREM sleep (a2), and REM sleep (a3) in *AVP-IRES-Cre* (AVP^DTA^; gray, *n* = 6 mice) and *VIP-IRES-Cre* (VIP^DTA^; gray, *n* = 6 mice) that received bilateral injections of DTA vector into the SCN, during the 4th and 5th day in constant darkness. **b** Upper panels: Amounts (mean ± s.e.m.) of the vigilance stages during the 4th and 5th subjective dark (SD4, SD5) and 4th and 5th subjective light (SL4, SL5) periods. Lower panels: 24 h amounts (mean ± s.e.m.) of the vigilance stages during the 4th and 5th day in constant darkness (DD4, DD5; lower left panels) and subjective night to subjective day (D/L) ratio for each vigilance stage during the 4th and 5th day in constant darkness (lower right panels). *n* = 6 mice per group,*p < 0.05, **, two-way ANOVA followed by a post hoc Bonferroni test. See also Figs. S7 and S8.

We next performed EEG analysis on the VIP^DTA^ mice to determine the effects of SCN^VIP^ cell loss upon on the circadian rhythms of sleep-wake (Fig. 4). Given the absence of LMA and Tb phenotypes in the AVP^DTA^ mice, and because they received the same vector injections, we used these mice as comparators. As compared to AVP^DTA^ mice, VIP^DTA^ mice exhibited a reduced circadian amplitude of the sleep-wake rhythm in DD. Selective ablation of SCN^VIP^ neurons did not produce significant changes in vigilance stage episode number or duration (Fig. S7). However, wakefulness and NREM sleep cortical EEG power circadian variations appeared phase-reversed across all frequency bands and at all vigilance stages (Fig. S8), resulting in significant differences in power variation at multiple time periods between AVP^DTA^ and VIP^DTA^ mice. These findings, in particular the observed reduction in circadian amplitude, are largely consistent to those in the *VIP*^*cre/cre*^*::Bmal1*^*fl/fl*^ mice, reinforcing the interesting possibility that SCN^VIP^ neurons may exert an influence on the expression of the sleep-wake rhythm, presumably by influencing the level of arousal or sleep.

### SCN^VIP^ and wheel running behaviors

**Fig. 5:**
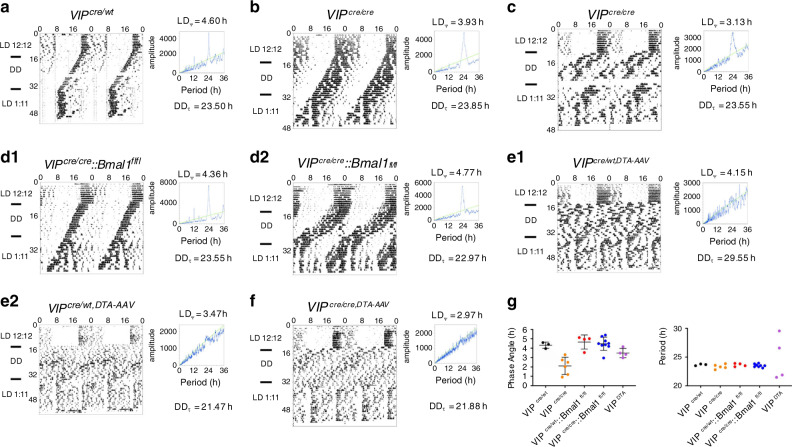
Circadian rhythms of wheel running behavior remain intact following loss of the molecular clock in SCN^VIP^ neurons, whereas their ablation disrupts this rhythm. **a**–**f** Wheel running behavior and associated periodograms from a control *VIP*^*cre/wt*^ mouse (**a**), two *VIP*^*cre/cre*^ mice (**b**, **c**), two *VIP*^*cre/cre*^*::Bmal1*^*fl/fl*^ mouse (**d**), two *VIP*^*cre/wt*^ mice that received bilateral injections of the DTA vector into the SCN (**e**) and a *VIP*^*cre/cre*^ mouse that received bilateral injections of the DTA vector into the SCN (F) under a 12:12 light-dark (LD, 10 days) cycle, in constant darkness (DD, 22 days), and under a 1:11 skeleton photo period (14 Days). Surprisingly, *VIP*^*cre/cre*^*::Bmal1*^*fl/fl*^ mice show intact rhythms of wheel running behavior following genetic deletion of *Bmal1* in SCN^VIP^ neurons. However, cell-specific ablation of SCN^VIP^ in *VIP*^*DTA*^ mice (both homozygous and heterozygous for the *VIP-Cre* gene) results in severe disruption of the wheel running rhythm. **g** Left, phase angle of entrainment for wheel running behavior in LD across all mice. (*VIP*^*cre/wt*^, *n* = 3; *VIP*^*cre/cre*^, *n* = 6; *VIP*^*cre/wt*^*::Bmal1*^*fl/fl*^, *n* = 4; *VIP*^*crecre*^*::Bmal1*^*fl/fl*^, *n* = 9; *VIP*^*DTA*^, *n* = 4). Right, period length in DD across all mice reveals that cell-specific ablations (VIP^DTA^) results in periods of wheel running behavior that, while disrupted, retain a circadian component. Means ± s.e.m. LD_Ψ_ = phase angle of entrainment, i.e., difference in hours between LD transition (ZT12) and rhythm acrophase. DDτ = free running period length.

We next evaluated the effects of (1) disruption of the molecular clock in SCN^VIP^ neurons, and (2) genetically driven ablation of SCN^VIP^ neurons on wheel running behavior in LD and DD. To do so, we generated new cohorts of mice and evaluated wheel running rhythms in LD, DD, and in a 1:11:1:11 skeleton photoperiod (SKP; Fig. 5a–e). In contrast to the arrhythmic LMA phenotype observed in the *VIP*^*cre/cre*^*::Bmal*^*fl/fl*^ mice under DD (see Fig. 1 b1-b2), wheel running behavior in DD was strikingly normal in *VIP*^*cre/cre*^*::Bmal*^*fl/fl*^ mice (Fig. 5d) as compared with *VIP*^*cre/wt*^ and *VIP*^*cre/cre*^ mice (Fig. 5a–b). Interestingly, however, we did observe mildly abnormal wheel running behavior in three (of seven) of our *VIP*^*cre/cre*^ mice, taking the form of an immediate advance (~2–4 h) of wheel running activity on transition from LD to DD and a lower amplitude/more fragmented circadian rhythm of wheel running activity in DD (Fig. 5c), which we view as additional evidence for a hypomorphic allele in the homozygous state. This phenotype was not observed in the remainder of our *VIP*^*cre/cre*^ or VIP^cre/wt^ controls, nor was it observed in any of the *VIP*^*cre/cre*^*::Bmal*^*fl/fl*^ mice (Fig. 5 d1-2), the latter suggesting that disruption of the molecular clock in SCN^VIP^ cells somehow compensated for the putative hypomophic phenotype observed in some of the *VIP*^*cre/cre*^ mice. Compared to VIP^cre/wt^ controls, which showed the expected unitary bout of wheel running activity under the 1:11:1:11 SKP, all of the *VIP*^*cre/cre*^*::Bmal*^*fl/fl*^ and *VIP*^*cre/cre*^ mice showed splitting (i.e., 2 daily bouts; Fig. 5b-d2) of their rhythms, which is consistent with that previously reported in VIP knockout (VIP^−/−^) and VIP receptor knockout (Vipr2^−/−^) mice^6^, and continues to point to a potential hypomorphic condition in the *VIP*^*cre/cre*^ mouse. On the other hand, and similar to our telemetry findings, wheel running rhythms in VIP^DTA^ mice during DD were severely disrupted. Despite appearance of arrhythmicity in several mice, all but one VIP^DTA^ mouse expressed a detectable circadian component in their wheel running rhythms, although the rhythms were low in amplitude and their period generally shorter than that of the other groups (Fig. 5 e1-f). Hence the genetically driven loss of SCN^VIP^ neurons resulted in a wheel running rhythm in DD that was similar, though not identical to that previously reported in the congenital VIP and VPAC2 KO models. Taken together, these findings suggest the interesting possibility that non-photic sensory flow during wheel running may provide an adequate forcing signal to ‘clockless’ VIP neurons (i.e., *VIP*^*cre/cre*^*::Bmal*^*fl/fl*^ mice*)*, resulting in the appearance of a consolidated wheel running rhythm. But in the absence of VIP neurons (i.e., VIP^DTA^ mice), and hence synaptic release of VIP, GABA or other diffusible factors used by SCN^VIP^ neurons, the SCN is compromised in its ability to convey these non-photic inputs to downstream intra- and extra-SCN effector circuits and hence produce a high-amplitude rhythm of wheel running activity. These results may also reflect a differential role for VIP in circadian function across development, as acute ablation in adulthood produces similar yet distinct phenotypes from genetic germ-line deletions. It is also worth mentioning that while wheel running behavior ostensibly provides another readout of SCN phase and period, at least one prior study has raised the concern that wheel running activity may not track with other physiological readouts of SCN phase, especially when animals have altered circadian phenotypes^26^. Our finding of similar, but also somewhat divergent not identical, effects of SCN^VIP^ manipulation on wheel running versus cage LMA is consistent with this prior suggestion. We opted not to perform a similar experiment with *AVP*^*cre/cre*^*::Bmal1*^*fl/fl*^ mice due to concerns that they were too sickly for the running wheel.

### Activation of SCN^VIP^ neurons does not drive sleep or arousal

**Fig. 6:**
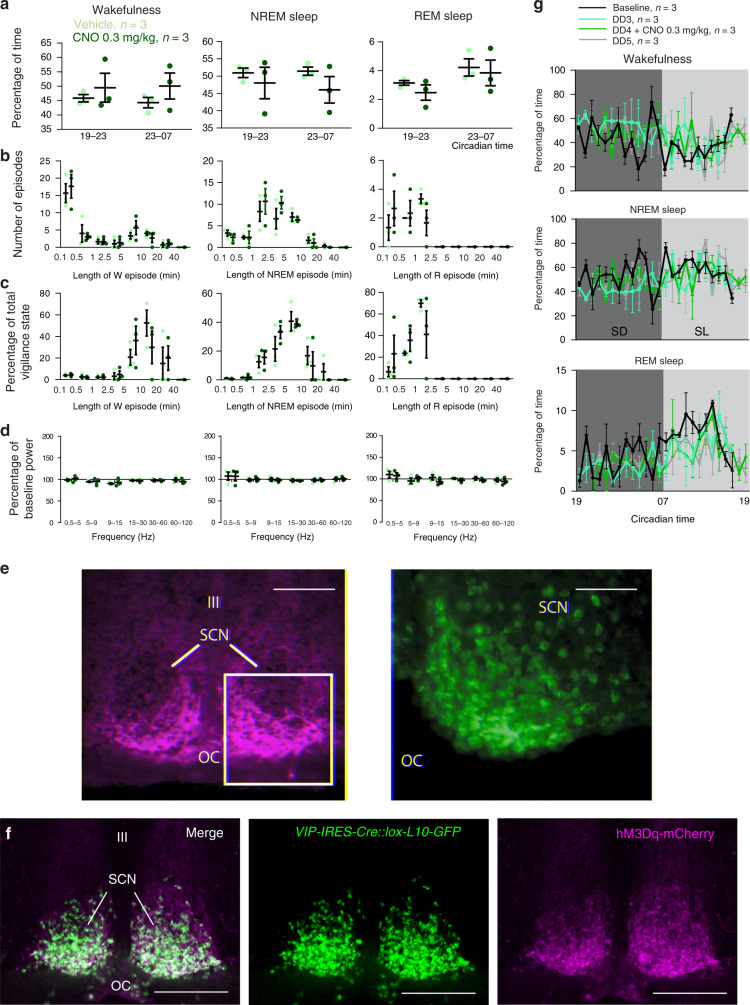
Chemogenetic activation of SCN^VIP^ neurons does not acutely promote sleep or wake. **a**–**d** Mice expressing the excitatory receptor hM3Dq in SCN^VIP^ neurons (*n* = 4) were injected with CNO (0.3 mg/kg, IP) or vehicle (saline) at 7 P.M. (ZT13). **a** Amount (mean ± s.e.m.) of the vigilance stages during the 4 h period following injections and of the remaining dark period. **b** Number of episodes (mean ± s.e.m.) of wake, NREM sleep or REM sleep binned in varying episode lengths and **c** time-weighted frequency histograms showing the percentage (mean ± s.e.m.) of wake, NREM sleep or REM sleep amounts binned for each episode length with respect to the total amount of wake, NREM sleep or REM sleep during the 4 h period following injections. **d** Cortical EEG power band changes from baseline. Delta (δ: 0.5–5 Hz), theta (θ: 5–9 Hz), sigma (σ: 9–15 Hz), beta (β: 15–30 Hz), low gamma (lγ: 30–60 Hz) and high gamma (hγ: 60–120 Hz) power band (mean ± s.e.m.) changes during the 3 h period following injections. **e** Left: Native mCherry fluorescence showing the AAV-hM3Dq-mCherry injection site in a *VIP-IRES-Cre::loxGFPL10* mouse (scale bar, 200 μm). Boxed area magnified and shown in the green color channel (right) illustrating robust expression of c-Fos in SCN neurons following administration of 0.3 mg/kg CNO (scale bar, 50 μm). **f** hM3Dq expression in the SCN of a *VIP-IRES-Cre::lox-L10-GFP* mouse, demonstrating that hM3Dq-mCherry was expressed eutopically. **g** Hourly amounts of wake, NREM sleep and REM sleep in baseline (LD) condition, during the 3rd day of constant darkness (DD3), following CNO (0.3 mg/kg) injection at 7 P.M., on the 4th day of constant darkness (DD4) and during the 5th day of constant darkness (DD5). **a**–**d**, **g** No significant difference, two-way ANOVA followed by a post hoc Bonferroni’s test. III, third ventricle; OC, optic chiasm.

Given the significantly blunted circadian amplitude of the sleep-wake cycle in both *VIP*^*cre/cre*^*::Bmal*^*fl/fl*^ and VIP^DTA^ mice, we asked whether acute and selective activation of SCN^VIP^ neurons might increase sleep or wake (Fig. 6). To do this, we placed bilateral injections of Cre-dependent hM3Dq into the SCN of heterozygous *VIP-ires-Cre* mice (*n* = 4) as previously described^27^, and administered the chemogenetic ligand clozapine-N-oxide (CNO; 0.3 mg/kg) at ZT4 (during the middle of the day when mice are typically less active) or ZT13 (1 h after lights off, when mice are typically most active). Strikingly, administration of CNO, which produced robust expression of c-Fos in mCherry+ (hM3Dq+) neurons, did not affect sleep-wake amounts (Fig. 6a), the number and length of vigilance state episodes (Fig. 6b), or their distribution (Fig. 6c) at ZT13. Similarly, no changes in the cortical EEG were observed in response to CNO administration (Fig. 6d). Finally, circadian sleep-wake distribution was not affected by activation of SCN^VIP^ neurons in DD condition (Fig. 6f) and the same results were obtained when CNO was injected at ZT4. These findings suggest that SCN^VIP^ neurons likely do not directly play a role in the modulation of behavioral state, i.e., not wake- or sleep-promoting per se. Rather, and instead, these findings inform the more parsimonious explanation that the blunted circadian amplitude of the sleep-wake rhythms observed in the *VIP*^*cre/cre*^*::Bmal1*^*fl/fl*^ mice and VIP^DTA^ mice were secondary to changes (i.e., arrhythmicity and fragmentation) of the LMA rhythm.

### Light but not behavioral state alters SCN^VIP^ cell activity

**Fig. 7:**
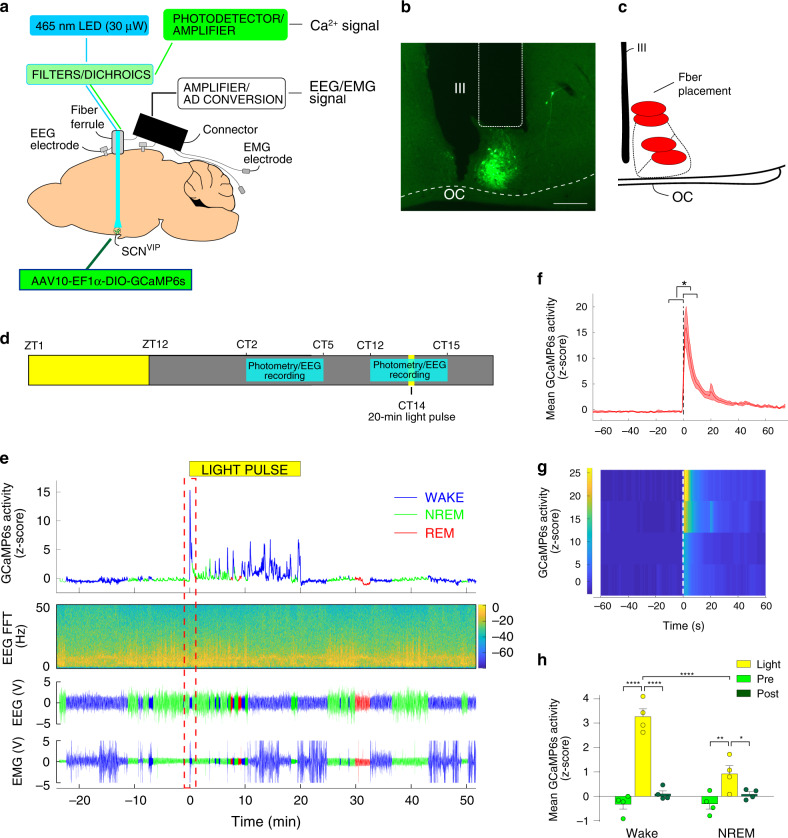
**In vivo** fiber photometry reveals SCN^VIP^ neurons are activated by light stimulation of the retina. **a** Experimental schema showing viral vector targeting and headset equipment for simultaneous in vivo fiber photometry and EEG/EMG recordings from mice expressing GCaMP6s in SCN^VIP^ neurons. **b** Representative photomicrograph of GCaMP6s expression in the SCN and location of the photometry optical fiber. Scale bar: 200 µm. OC; optic chiasm, III; 3rd ventricle. **c** Schematic of SCN area showing fiber placement from experimental mice over the SCN. **d** Behavioral experimental paradigm showing times of photometry/EEG recording related to the time of day and the light pulse stimulus. **e** Representative example showing GCaMP6s signal (upper panel), EEG frequency spectrogram (2nd panel from top), raw EEG (3rd panel from top) and raw EMG (bottom panel) before, during and after the 20-min light pulse at ZT14. Red dotted rectangle indicates time displayed in (**f**, **g**). **f** Mean GCaMP6s activity over all mice at the initiation of the light pulse. Black dotted line indicates onset of the light pulse. Shaded area indicates s.e.m. Paired *t*-test (two-tailed) * <0.0206. *n* = 4. **g** Heatmap depicting GCaMP6s activity at the light pulse for each mouse. White dotted line indicates onset of the light pulse. **h** Mean (+ s.e.m.) GCaMP6s activity during wake and NREM sleep before, during and after the light pulse. RM two-way ANOVA (Interaction: Light Pulse x Arousal State F (2, 6) = 62.82, *p* < 0.0001; *n* = 4 followed by Sidak’s post hoc tests (*p < 0.05, **p < 0.01, ****p < 0.0001)). See also Fig. S9.

To help reconcile the observed reduction in the amplitude of the sleep-wake cycle in the *VIP*^*cre/cre*^*::Bmal1*^*fl/fl*^ mice and VIP^DTA^ mice with the contrasting absence of effect of acute activation of SCN^VIP^ neurons on sleep or wake, we next examined the state-dependent population Ca^2+^ activity dynamics of SCN^VIP^ neurons using in vivo fiber photometry (Fig. 7). To do so, we placed unilateral injections of DIO-GCaMP6s-AAV10 into the SCN of *VIP*^*cre/wt*^ mice and implanted a photometry fiber immediately dorsal to the SCN^VIP^ cell bodies (Fig. 7a–c). Four-six weeks after injections of the viral vector, mice were habituated to the recording conditions before being placed in DD at ZT12. After the mice were released into DD, on the next day, simultaneous sleep-wake and photometry recordings were carried out at CT 2–5 and CT 12-15 with a 20-min light pulse applied at CT14 (i.e., 26 h after onset of DD, Fig. 7d). SCN^VIP^ neurons were immediately activated by the light pulse (Fig. 7e–g), indicating that light stimulation at the retina results in robust excitation of the SCN^VIP^ cell population. As an important control, VIP-containing neurons in the rostrally adjacent ventromedial preoptic nucleus (VMPO) were not activated by the light pulse (Fig. S9 A-B), indicating that neuronal activation by light is selective to SCN^VIP^ neurons and not simply an artifact of external light pollution. Following the initial peak induced by the light pulse, Ca^2+^ activity declined to baseline levels. Interestingly, further Ca^2+^ peaks were observed throughout the remainder of the light pulse when the mice were awake, but not when they were in NREM or REM sleep (Fig. 7e). These peaks in Ca^2+^ activity were only observed during the 20-min light pulse and did not occur during waking episodes either prior to, or immediately after the cessation of, the light pulse (Fig. 7h), nor were they present during wake in the absence of light—hence this was not a movement artifact. Apart from the activity observed during the light pulse, the activity profile of SCN^VIP^ neurons did not fluctuate significantly over sleep-wake states or at arousal state transitions, and this was independent of the time of day (Fig. S9C-I). In sum our findings show that the SCN^VIP^ population is highly responsive to light input in vivo, which is congruent with a recent report^28^, but also that their activity is behavioral state independent. The latter observation is consistent with our chemogenetic findings which suggest that SCN^VIP^ neurons may not contribute directly to the regulation of sleep-wake architecture, timing or amounts.

### Mapping functional synaptic outflow of SCN^VIP^ cells

While the foregoing work points to an indispensable role for SCN^VIP^ neurons in producing coherent circadian rhythms of LMA, the post-synaptic targets by which SCN^VIP^ neurons drive the LMA rhythm (or nearly any output rhythm) remain incompletely understood. To initially map efferent projections of SCN^VIP^ neurons we placed unilateral injections of a genetically encoded anterograde tracer into the SCN of *VIP*^*cre/wt*^ mice. As shown in Fig. S10, intra-hypothalamic and preoptic regions were primary targets of SCN^VIP^ neurons. The presumptive terminal fields within the dorsomedial hypothalamus (DMH) were of particular interest given previous lesion studies that identified an important role for the DMH in the expression of the LMA circadian rhythm^29^. The density of SCN^VIP^ terminal fields within the DMH was nevertheless rather modest, suggesting that a portion of SCN^VIP^ inputs to the DMH may be indirect (polysynaptic). To this end, prior lesion studies have suggested an important role for a polysynaptic pathway, involving the SPZ, in regulating the circadian rhythm of LMA^29,30^. We therefore asked whether SCN^VIP^ neurons are functionally, synaptically coupled with DMH neurons via the SPZ. Indeed, a SCN → SPZ → DMH circuit has been previously proposed^29,31^, but never functionally confirmed.

**Fig. 8:**
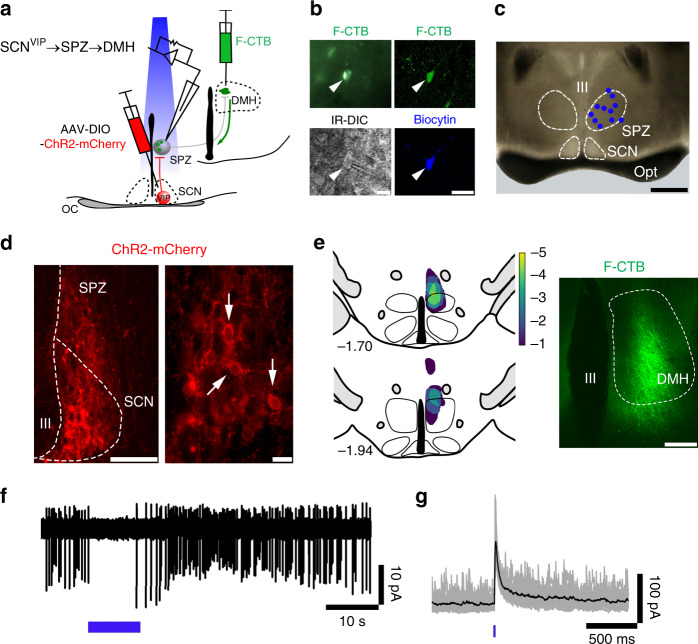
SCN^VIP^ neurons inhibit SPZ neurons that project to the DMH (SCN^VIP^ **→** SPZ **→** DMH). **a** Schematic of the experimental design used to map SCN^VIP^ → SPZ → DMH connectivity in brain slices. *AAV-DIO-ChR2-mCherry* was injected into the SCN of *VIP*^*cre/wt*^ mice and in the same animals, green-fluorescent CTB (F-CTB; Alexa Fluor-488 conjugated-CTB) was injected into the ipsilateral DMH, to retrogradely label SPZ neurons that project to the DMH. Recordings were made from SPZ neurons labeled with F-CTB in green while photostimulating SCN^VIP^ axon terminals in the SPZ. **b** Examples of SPZ neurons retrogradely labeled (upper panels) from the DMH and imaged during whole-cell recordings under fluorescent and IR-DIC visualization (lower left) or after post hoc labeling of biocytin with streptavidin-conjugated AlexaFluor-405 (blue; lower right). Scale bar: 20 µm). **c** Map of 11 recorded SPZ DMH projecting neurons identified after post hoc labeling of biocytin (scale bar: 500 µm). **d** Photomicrograph, illustrating ChR2-mCherry expression in SCN^VIP^ neurons (marked by arrows) at low (left; scale bar: 100 µm) and higher (right; scale bar: 20 µm) magnifications. **e** Heatmap of the distribution of F-CTB in the DMH in five recorded mice (*left*) and an example of green F-CTB in the DMH (right; scale bar: 200 µm). **f**, **g** Photostimulation of SCN^VIP^ input inhibited action potential firing of SPZ DMH projecting neurons (**f** cell-attached recording, blue bar represents a 10-s train stimulation at 10 Hz; 10 ms light pulses) and in the same neuron in whole-cell configuration, elicited photo-evoked IPSCs (**g** blue bar represents a 10-ms light pulse; V_h_ = 0 mV). Individual photo-evoked IPSCs are shown in gray and the average trace in black. OC: optic tract; III third ventricle.

To assess functional polysynaptic connectivity, we first tested whether SCN^VIP^ neurons inhibit SPZ neurons that project to the DMH (SCN^VIP^ → SPZ → DMH) by performing Channelrhodopsin(ChR2)-assisted circuit mapping (CRACM) in brain slices, as previously described^32,33^. We expressed ChR2-mCherry in SCN^VIP^ neurons by injecting AAV-DIO-ChR2-mCherry into the SCN of *VIP*^*cre/wt*^ mice. In the same mice, we placed injections of green-fluorescent CTB (F-CTB) into the ipsilateral DMH. Five to six weeks later, we recorded from SPZ neurons that were retrogradely labeled from the DMH while photostimulating fibers and terminals from SCN^VIP^ neurons in SPZ (Fig. 8a). Of the 20 recorded SPZ-DMH projecting neurons, we labeled 11 of these neurons with biocytin and confirmed that they were located in the SPZ (Fig. 8b–c). Histological assessment confirmed that expression of ChR2-mCherry and F-CTB were restricted to the SCN and DMH, respectively (Fig. 8d–e). Photostimulation of the SCN^VIP^ → SPZ input inhibited action potential firing, and evoked inhibitory postsynaptic currents (IPSCs) in 6 out of 20 SPZ → DMH projecting neurons (Fig. 8f–g). The probability of a connected cell responding to photo-stimulation was 97.78 ± 1.65%, the average photo-evoked IPSC peak amplitude was 107.20 ± 26.88 pA and the photo-evoked IPSC latency was 9.97 ± 0.84 ms. These findings confirm that SCN^VIP^ cells are functionally synaptically coupled, via a cellular SPZ relay, with DMH cells and identify a circuit substrate by which SCN^VIP^ neurons may regulate LMA rhythms. These results also re-emphasize the functional and anatomical importance of the cellular SPZ as a postsynaptic target of, and relay for, SCN neurons and their synaptic outflow^33–35^.

### Photic and non-photic presynaptic inputs to SCN^VIP^ cells

**Fig. 9:**
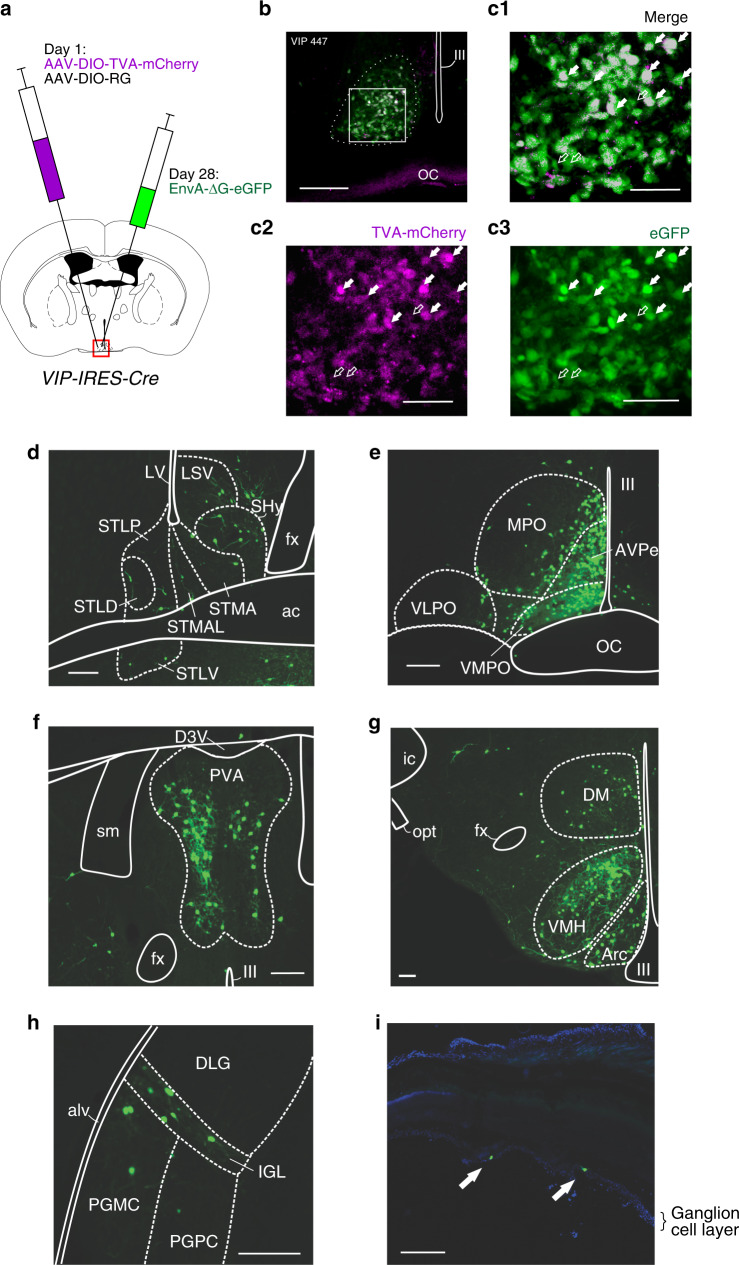
Presynaptic inputs to SCN^VIP^ neurons arise from areas involved in photic and nonphotic sensory regulation of the SCN clock. **a** Schematic of experimental procedure. AAV8-DIO-TVA-mCherry and AAV8-DIO-RG were co-injected into the SCN of *VIP-IRES-Cre* mice at Day 1. 28 days later, the pseudotyped modified rabies virus (EnvA-ΔG-eGFP) was injected at the same site in the SCN and the mice perfused 11 days later. **b** Photomicrograph of the SCN illustrating AAV8-DIO-TVA-mCherry expressing neurons (magenta) and EnvA-ΔG-eGFP expressing neurons (green). Co-expression of AAV8-DIO-TVA-mCherry and EnvA-ΔG-eGFP represents the starter population. Scale bar: 200 µm. (**C1–3**) High magnification image of box in B (C1), red channel only (C2) and green channel only (C3). Filled arrows point towards starter population neurons expressing both AAV8-DIO-TVA-mCherry and EnvA-ΔG-eGFP. Unfilled arrows point towards neurons expressing EnvA-ΔG-eGFP only (retrogradely labeled neurons). Scale bar: 100 µm. **d**–**h** Photomicrographs showing SCN^VIP^ brain input populations in: LS, BNST and SHy (**d**); VMPO, MPO, AVPe (**e**); PV (**f**); Arc, VMH, DMH (**g**); IGL and PH (**h**). Scale bar: 200 µm. **i** Photomicrograph of retina showing EnvA-ΔG-eGFP expressing neurons (green, filled arrows), counter-stained with DAPI. Scale bar: 200 µm. Also see Fig S10. For abbreviations and quantitative proportion of inputs from regions throughout the brain, see Supplementary Information.

While previous studies using conventional tracing techniques have identified inputs to the SCN, these approaches have lacked specificity, particularly with respect to defining afferents to specific SCN cell types, including SCN^VIP^ neurons. We therefore sought to determine the extra-SCN sources of synaptic inputs to SCN^VIP^ neurons using conditional monosynaptic retrograde rabies tracing (Fig. 9a). As shown in Fig. 9b, c, the starter population of VIP neurons was restricted to the SCN. Many retrogradely labeled neurons were observed in the lateral septum (LS), the bed nucleus of the stria terminalis (BNST), the septohypothalamic nucleus (SHy), the medial preoptic (MPO), the VMPO, the anteroventral periventricular nucleus (AVPe), the paraventricular thalamic nucleus (PVA), the ventromedial hypothalamic nucleus (VMH), the arcuate nucleus (ARC), the DMH, the intergeniculate leaflet (IGL) and scattered throughout the retina (Fig. 9d–j). Our findings are broadly consistent with other studies describing afferents to the SCN in the hamster^36^, rat^37,38^, and mouse^39^. However, and unlike these previous studies, we did not observe retrogradely labeled neurons in the hippocampus or dorsal raphe nucleus. Altogether, our results therefore confirm and extend previous anatomic studies in establishing SCN^VIP^ as post-synaptic targets of key brain structures involved in mediating photic and non-photic influences on the clock.

### SCN^VIP^ neurons comprise two molecularly distinct subtypes

**Fig. 10:**
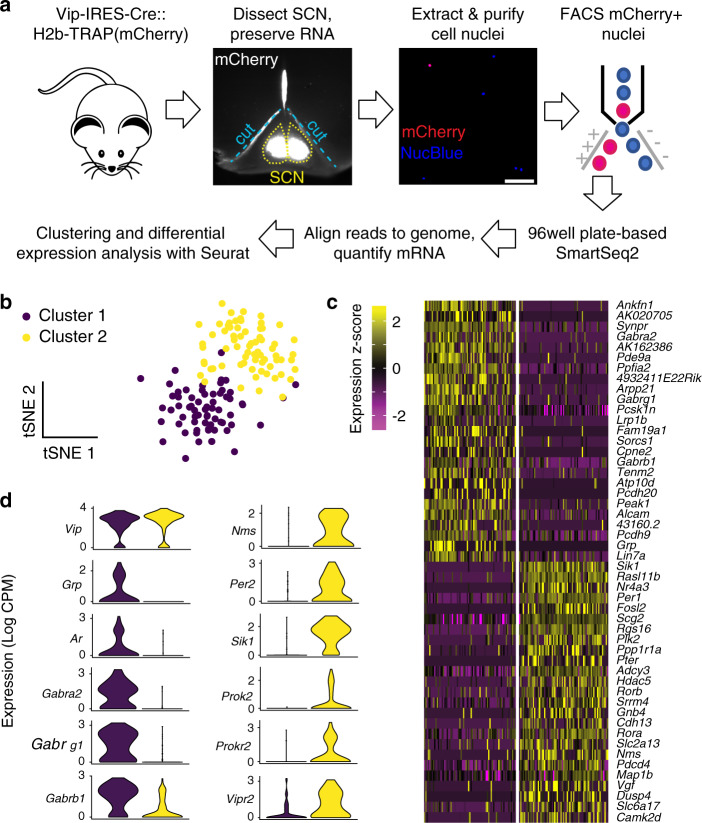
sNuc-seq analysis reveals two transcriptionally distinct populations of SCN^VIP^ neurons. **a** Schematic of method for labeling, isolating, and profiling SCN^VIP^ neuron transcriptomes for sNuc-Seq; images are from samples used in this study. **b** t-Distributed Stochastic Neighbor Embedding (tSNE) plot of 144 VIP SCN neurons (dots) profiled by sNuc-Seq and clustered according to their expression of 689 high-dispersion genes; colors indicate cluster identity. **c** Heatmap showing single-cell (column) expression of top cluster marker genes (rows), with cells (columns) binned by cluster identity. **d** Violin plots of single-cell expression values of *Vip* and selected cluster marker genes. See also Fig. S11.

Previous studies in rodent models have raised the possibility that SCN^VIP^ neurons are a heterogenous population containing anatomically and physiologically distinct subtypes^40,41^. However, the molecular identity of these subtypes and the full extent of their differences remain unclear. We therefore used sNuc-Seq^42^, a systematic means of classifying cell types based on the nuclear mRNA profiles of single cells, to characterize the molecular diversity of SCN^VIP^ neurons. First, to label VIP neuron nuclei for sampling, we crossed *VIP-IRES-Cre* mice to a transgenic reporter mouse line that Cre-dependently expresses a nuclear-localized mCherry (H2b-TRAP)^43^. We then performed sNuc-Seq on mCherry+ cell nuclei sorted from juvenile and adult male *VIP-IRES-Cre::H2b-TRAP* SCN tissue that had been dissected one hour after light cycle onset (Fig. 10a). We sequenced each of 192 single-nuclei samples to a depth of 3.0 M ± 1.4 M reads per sample, aligned the reads to the mouse genome (mean and S.D. reads aligned per sample, 79% ± 22%), and quantified expression values based on the number of reads aligning to each gene (see “Methods”). After filtering out the 7% of samples in which fewer than 500 genes were detected, we used the remaining 178 single-nuclei transcriptomes, averaging (±S.D.) 3694 ± 1425 genes each, for unsupervised clustering and differential expression analysis.

Our initial results showed three clusters of VIP neurons (Fig. S11A). Each cluster contained cells from each age group (Fig. S11A), indicating that the transcriptional identities of these groups are stable across late development and thereby justifying our pooling of the data for analysis. However, only two of the three initial clusters were confirmed as SCN neurons based on their expression of the regional marker genes, *Lhx1* and *Vipr2*^44,45^ (Fig. S11B) and so were the focus of our subsequent analysis.

Re-clustering only the neurons that contained SCN marker genes revealed two subtypes of SCN^VIP^ neurons with distinct transcriptional profiles (Fig. 10b, c). We annotated these two subtypes as *Grp*^VIP^ neurons and *Nms*^VIP^ neurons based on their enriched expression of the neuropeptide-encoding genes. These subtypes are consistent with the two VIP neuron subtypes, similarly called Vip/Grp neurons and Vip/Nms neurons, identified by a study published during the revision of this manuscript (Fig. S11C). Contrasting their transcriptional profiles revealed gene sets which are consistent with distinct functional roles. For instance, consistent with a central role in circadian clock function, *Nms*^VIP^ neurons were enriched with transcripts encoding components and regulators of the circadian clock, including: *Per2*; salt inducible kinase 1 (*Sik1*)^46^; *Prok2*^47^, and *Prokr2*^48^. On the other hand, *Grp*^VIP^ neurons had higher expression of GABA receptor genes (*Gabra2*, *Gabrg1*, *Gabrb1*) and androgen receptors (Ars) and therefore *Grp*^VIP^ SCN neurons may be an important cellular point of entry to the SCN clock for non-photic inhibitory inputs, including androgens that can potently alter the circadian period^49,50^.

### Technical consideration: a hypomorphic allele?

While our physiological and behavioral recordings in the *VIP*^*cre/cre*^ mouse are potentially consistent with a hypomorphic allele, the expression of *VIP* transcript (per RNAscope in situ hybridization) and number of VIP+ cell bodies (per GFP+ cells in the genetic cross) within the SCN did not appear to be appreciably altered in the homozygous state. It is nevertheless possible, however, that less VIP was being produced in the SCN of the homozygous *VIP*^*cre/cre*^ mouse, as has been reported before in other targeted transgenic IRES-Cre mouse lines^21^, and as was clearly the case for the Vip-IRES-Cre (JAX #010908) mouse line that has been employed in a number of previous chronobiology studies^51^. If it were the case that less VIP was produced in the SCN of our mice, however, the mild phenotypes observed in the hypomorph would only emphasize the importance of VIP in proper SCN network function (i.e., other SCN cell populations are apparently unable to fully compensate for the presumptive decrease in VIP production). As another consideration, SCN^VIP^ neurons co-express and release GABA, and as such it is possible that GABA-VIP synaptic “balance” may have been disrupted in the *VIP*^*cre/cre*^ mouse and thereby contributed to the phenotypes (e.g., high variability in the phase angle of entrainment of Tb and LMA). To begin to explore this interesting possibility, we selectively disrupted GABAergic signaling by VIP neurons to see if this would “rescue” the mild circadian phenotype observed in our *VIP*^*cre/cre*^ mice. To do so, we generated *VIP*^*cre/cre*^*::Vgat*^*fl/fl*^ mice. Rather remarkably, we found that disruption of GABAergic signaling by VIP neurons in the *VIP*^*cre/cre*^ mouse (putative hypomorphic) reduced the variability in the LD phase angle of entrainment for Tb, but not for LMA (Fig. S12). While a finding that could have explanatory power for the previously reported “desynchronizing” effects of SCN GABA as well as implications for the molecular basis of entrainment, future intersectional studies will be needed to better understand the functional relationship between GABA and VIP synaptic release by SCN^VIP^ and their individual roles in SCN circuit function.

## Discussion

Altogether considerable evidence has accumulated pointing to an important role for VIP, and its cognate receptor, VPAC2, in SCN clock function, yet more recent studies have suggested that other, non-VIP SCN cell groups may play a more dominant role in circadian pacemaking. Here we show that SCN^VIP^ neurons, and their internal molecular clock, are in fact necessary for establishing and maintaining coherent circadian rhythms of LMA, and for the normal expression of the circadian rhythms of Tb, sleep-wake and wheel running behavior. While our findings do not exclude the possibility that SCN^VIP^ neurons may also directly contribute to the regulation of the sleep-wake cycle, both the absence of an effect of acute chemogenetic activation of SCN^VIP^ neurons on sleep-wake, and our finding that the activity dynamics of SCN^VIP^ neurons are behavioral state invariant, instead support the null hypothesis. Regardless, our in vitro CRACM experiments establish the presence of a functional SCN^VIP^ → SPZ → DMH synaptic pathway by which these neurons may regulate circadian rhythms of LMA. We also found that SCN^VIP^ neurons are post-synaptic targets of key structures mediating non-photic and photic modulation of the SCN clock and hence likely comprise a cellular point of integration of these inputs. Finally, our sNuc-seq results reveal that a subset of SCN^VIP^ neurons, *Nms*^VIP^ neurons, express core components and regulators of the circadian clock, inferring pacemaker potential, and that another subset of SCN^VIP^ neurons, *Grp*^VIP^, more uniquely expresses high levels of GABA and Ars and hence may function as cellular effectors for non-photic inputs, including gonadal hormones.

In the present study, we found that developmental disruption of the molecular clock within SCN^VIP^ neurons produced, in DD, arrhythmicity in LMA and altered the expression of the Tb and sleep-wake rhythms. Selective, genetically driven ablation of SCN^VIP^ neurons in adult mice recapitulated the arrhythmic LMA phenotype but also profoundly altered the expression of Tb and wheel-running activity rhythms. With respect to other established pacemaker cell populations, seminal work by Lee and colleagues found that a subset of SCN neurons expressing NMS (~45–50% of total SCN neuronal population) plays an essential role in the generation of daily rhythms in behavior^11^. These authors specifically showed that disruption of the molecular clock in NMS neurons produces arrhythmic wheel running activity and, moreover, that NMS neurons control in vivo circadian rhythms through intercellular synaptic transmission. Our data suggest the possibility that the arrhythmic phenotype observed in the NMS mice experiments may link to disruption of the *Nms*^VIP^ population. The fact that non-VIP NMS, including *Grp*^VIP^ or AVP, cells were unable to compensate for the absence of *Nms*^VIP^ neurons, underscores this point. Our findings are moreover consistent with recent findings by two different groups who reported that activation or inhibition of VIP cells in vivo can acutely modulate LMA rhythms^40,52^.

In a recent study Jones et al.^28^ showed, using in vivo fiber photometry, that SCN^VIP^ neurons exhibit circadian rhythms of spontaneous activity, are acutely responsive to light, and are essential for the normal resetting of daily rhythms by environmental light. Using a similar approach, but here in combination with chronic EEG recordings, we also found that SCN^VIP^ cells are highly responsive to light, but that their activity is otherwise behavioral state invariant. During a light pulse administered at CT14 we were surprised to observe additional Ca^2+^ peaks that occurred selectively when mice were awake but not when they were in NREM or REM sleep. It is thus tempting to speculate that the Ca^2+^ activity peaks uniquely observed during the light pulse and waking condition reflect integration of non-photic (e.g., locomotor, head movement or other signals associated with wake) and photic sensory flow by SCN^VIP^ neurons^53,54^. This hypothesis derives additional support from our anatomic findings that revealed inputs to SCN^VIP^ neurons from both the retina and the IGL, the latter structure long-implicated in non-photic resetting of the clock^55^. Put another way, our anatomic findings show that SCN^VIP^ neurons can be presynaptically modulated by both photic and non-photic sensory flow, and taken together with our photometry findings, suggest the interesting possibility that SCN^VIP^ neurons may actually represent a key cellular point of integration of these sensory inputs. To this end, we also noted substantial input from several preoptic cell groups, suggesting a potential circuit substrate by which temperature sensitive neurons might modulate, and possibly entrain, SCN^VIP^ neurons^56^. It is also useful to contrast our findings with those from a recent rabies-based tracing study^57^ which showed that CCK-expressing SCN neurons, which are generally intermingled with, but are also distinct from, SCN^VIP^ neurons, receive input from a large number of hypothalamic and preoptic structures, but not from the retina, and minimally from the IGL.

Our findings of molecular heterogeneity within the SCN^VIP^ cell population considerably extend findings from previous studies. For example, Kawamoto et al.^41^ demonstrated that SCN^VIP^ neurons can be divided into two groups based on light-inducibility of the *Per1* gene, innervation of retinal afferents, day-night variation in VIP mRNA, and the coexpression of GRP. Similarly, Mazuski et al.^40^ found two classes of instantaneous firing activity in SCN^VIP^ neurons. Our finding that *Sik1* is primarily expressed in *Nms*^VIP^, i.e., GRP-negative subtype, of SCN^VIP^ neurons, provides an important functional-anatomical framework for the findings by Jagannath et al.^46^ who showed that *Sik1* and CREB-regulated transcription coactivator 1 (CRTC1) are involved in clock resetting. In addition, Model et al.^58^ had previously shown that gonadectomy influences circadian period by acting directly on the SCN, and that Ars are located in the ventrolateral SCN^49^. While the cell type expressing the Ars remained unclear, localization to SCN GRP-positive cells has been suggested^49^. Our transcriptional data confirm that Ars are localized to the *Grp*^VIP^ cells and provides additional evidence of the potent influence that even a subset of SCN^VIP^ neurons can have on ensemble period, although an additional contributing influence of non-VIP GRP SCN neurons that also harbor Ars cannot be discounted. It has also been shown that SCN photic sensitivity can be modulated by changes in the endocrine milieu^49,50^, which provides yet another compelling indication that SCN^VIP^/*Grp*^VIP^ neurons integrate photic and non-photic inputs to modulate the output of the SCN clock. Our transcriptional profile of *Grp*^VIP^ SCN neurons also raises the possibility that GRP-VIP neurons are non-circadian as they lack detectable clock gene expression. While it is possible that *Grp*^VIP^ neurons express circadian genes at other time points or under other conditions, the depth of sequencing we achieved would argue against our negative finding reflecting a failure of detection. Interestingly, a lack of detectable rhythmicity in clock gene expression in SCN GRP cells was previously reported^59^. Hence it is unlikely that *Grp*^VIP^ SCN neurons possess endogenous circadian pacemaker potential, but rather are entrained through coupling to SCN *Nms*^VIP^ pacemaker cells. Our sNuc-Seq findings also lend additional support to the surmise that the phenotypes observed in the NMS mice link, at least in part, to disruption of the *Nms*^VIP^ population, given that this subpopulation was enriched with transcripts encoding components and regulators of the circadian clock. Finally, the finding of molecular heterogeneity provides another example of apparent conserved features between VIP and pigment dispersing factor (PDF), which is a neuropeptide related to VIP that is found in insect circadian systems and whose cell bodies appear to be comprised of two distinct subpopulations^60,61^.

While SCN^VIP^ neurons comprise only ~10% of the total cellular SCN, our findings indicate an indispensable role for this cell population in the regulation of the LMA rhythm as well as for the normal expression of the Tb, sleep-wake and wheel-running rhythms. Specifically, we found that other neuropeptide systems in the SCN network, in either the acute or developmental framework, were unable to compensate for the loss of SCN^VIP^ neurons, or their cellular clocks, to maintain coherent LMA rhythms or high-amplitude Tb and sleep-wake rhythms. Given differential disruptive effects of our SCN^VIP^ cell manipulations on LMA, Tb, sleep-wake, and wheel running rhythms, our findings also lend support to a model in which neurochemically defined SCN cell groups utilize differential synaptic outflow pathways and downstream circuit targets to impart circadian timing information on a wide range of physiological and behavioral processes. This model derives additional support from a recent paper by Paul and colleagues who showed that selective manipulation of SCN^VIP^ cells can acutely modulate cardiovascular function, likely via direct synaptic inputs to pre-autonomic neurons within the dorsal cap of the PVH (a region we also found to be a dense post-synaptic target of SCN^VIP^ cells—see Fig. S10)^52^. Our results further align with findings in aging humans, wherein the LMA, rhythm is disrupted in association with loss of SCN^VIP^ neurons^62^ and also with recent findings linking a polysynaptic SCN^VIP^→VMH circuit with rhythms of aggression^33^.

The present study also provides a template for future experimental interrogation of the cellular SCN, including intersectional approaches. As examples, such approaches would help address clear knowledge gaps that exist with respect to (1) molecular heterogeneity within other canonical SCN cell populations (e.g., AVP, NMS), (2) how temporal information (phase) is synaptically communicated to downstream effector networks, (3) the behavioral state activity dynamics of different SCN cell populations, and (4) how the various SCN cell populations, including functional subpopulations, are regulated synaptically by sensory flow to modulate fundamental properties of the circadian clock such as phase, amplitude and period. Hence, for example, future CRACM work using other Cre-driver mouse lines, including the development of newer lines informed by cell-type specific transcriptomics^63^, will facilitate the development of a comprehensive map of SCN synaptic outflow. In turn this will enable a more detailed understanding of the circuit and synaptic bases by which various SCN cell populations impart temporal organization on a broad range of physiological and behavioral outputs.

## Methods

### Generation and validation of *VIP-IRES-Cr*e mice

*VIP-IRES-Cre* mice were generated using standard recombineering techniques^14^. In brief, a selection cassette containing an optimized internal ribosomal entry sequence linked to Cre recombinase and a Frt-flanked kanamycin resistance gene was targeted 3 bp downstream of the stop codon of the VIP gene in a bacterial artificial chromosome (RP24-216N8; Children’s Hospital Oakland Research Institute). A targeting plasmid containing the Cre-containing selection cassette and ~4 kb genomic sequence upstream and downstream of the Vip stop codon was isolated and used for embryonic stem cell targeting by the Beth Israel Deaconess Transgenic Core. Correctly targeted clones were identified by long range PCR. Chimeric animals generated from blastocyst implantation were then bred for germline transmission of the *VIP-IRES-Cre* allele. Flp-deleter mice were then used to remove the neomycin selection cassette. Mice were subsequently intercrossed to generate homozygous Cre reporter strains and bred to the *R26-loxSTOPlox-L10-GFP* reporter line^64^. Results from the reporter cross indicated that expression of the transgene was restricted to the ventrolateral (core) SCN (Fig. 1a). In the *VIP-IRES-Cre::L10-GFP* cross we also confirmed the presence of a previously described population of VIP neurons in the preoptic region, ~1 mm rostral to the SCN. Given the relative proximity of the preoptic and SCN VIP populations, we constrained the volumes of our AAV injections targeting the SCN to avoid concurrent transfection of the preoptic VIP cell population. We subsequently confirmed that the preoptic VIP population is transcriptionally unique from the SCN VIP cell population and, per fiber photometry, exhibits markedly different population cell activity dynamics across the 24 h day and does not respond to an administration of a light pulse (see Fig. S9).

### Stereotaxic brain injections

For selective ablation of SCN^VIP^ and SCN^AVP^ neurons, *VIP-IRES-cre* and *AVP-IRES-Cre* mice received bilateral injections of AAV10-hSyn-mCherry-DIO-DTA (DTA-AAV; 30-40 nl per side, acquired from PMF) into the SCN. The DTA vector was created by Drs. Patrick Fuller and Michael Lazarus, has undergone extensive validation, is published^21,22^, and is freely available upon request (as plasmid or AAV). Following injections of DTA-AAV, and per prior validation studies with this viral-based toxin, we allowed at least 4 weeks for the lesion to develop before starting the LD portion of our recordings.

For chemogenetic experiments, AAV10-EF1α-DIO-hM3Dq-mCherry (30-40 nl per side; acquired from PMF^27^, was injected into the bilaterally into the SCN in *VIP-IRES-Cre mice*.

For ChR2-assisted circuit mapping (CRACM) experiments, we injected *VIP-IRES-Cre* mice (*n* = 5) unilaterally with 30 nl of AAV-DIO-ChR2-mCherry (UNC vector core) and placed a second injection of 10 nl of Fluorescent-conjugated Cholera Toxin Subunit B (F-CTB; green Alexa Fluor-488 conjugated-CTB; Invitrogen) into the ipsilateral DMH of the same mice, to retrogradely label SPZ neurons projecting to the DMH.

For photometry experiments, AAV10-EF1α-DIO-GCaMP6s (40 nl; acquired from PMF^65^ was injected into the left SCN in male *VIP-IRES-Cre* mice.

For monosynaptic retrograde modified rabies tracing we first made injections of a mixture of 60 nl AAV8- EF1ɑ -DIO-TVA (UNC vector core) and 60 nl AAV8-CAG-DIO-RG (GVVC-AAV-59, Stanford vector core) into the left SCN. Four weeks after the initial surgeries, 200 nl of EnvA-ΔG-eGFP (Salk Institute) was injected into the same place.

In brief, mice were anesthetized with a ketamine/xylazine mixture (100 and 10 mg/kg, respectively, IP) and administered slow release meloxicam subcutaneously (4 mg/kg) for pain relief. Fur was removed from the top of the skull with clippers and the area sterilized with iodopovidone, followed by isopropyl alcohol. The mouse was secured in a stereotaxic frame and an incision was made down the midline of the skull and the skin was retracted to expose bregma. A small burr hole was drilled through the skull above the SCN and a fine glass pipette containing the viral vector was slowly lowered down to the SCN (AP: −0.4 from Bregma, DV: −5.0, ML: 0.3, using coordinates derived from the atlas of Paxinos and Franklin)^66^ using a Kopf high precision micro-manipulator. Viral vector was dispelled slowly at a rate of <1 nl/second using a pico-spritzer air puffer system. Once the required volume of virus had been dispelled, the pipette was left in position for 3 min before being slowly raised out of the brain. For CRACM experiments, a second injection of F-CTB was made into the DMH (AP: −1.6 mm, DV: −3.5 mm, ML: −0.2 mm) during the same surgery. The skin was sutured back together and 0.5 ml sterile saline administered subcutaneously before the mouse was allowed to resume consciousness on a heating pad.

### Biotelemetry implantations and recordings

Mice were anesthetized with ketamine/xylazine [100 and 10 mg/kg respectively, intraperitoneal (IP)]. An incision was made in the IP cavity and biotelemetry transmitters (ETA-F10, Data Sciences International, St. Paul, MN) were implanted. Incisions were sutured and treated by topical antibiotic. After at least two weeks of implantation, mice were housed in standard cages placed atop a biotelemetry receiver, interfaced to a microcomputer data acquisition system (Data Sciences International), in an insulated sound-proofed recording chamber maintained at an ambient temperature of 22 ± 1 °C and on a 12 h light 12 h dark cycle (lights-on at 7 A.M., Zeitgeber time: ZT0) with food and water available ad libitum. Mice were habituated to these chambers for at least three days before recording. Body temperature values were recorded at 5-min intervals, and LMA data were collected in 5-min bins. Mice were recorded for at least 10 days under an LD cycle, followed by at least 3 weeks in DD, and then returned to LD conditions for at least 7 days. LMA and Tb data were first analyzed and plotted using Clocklab (Coulbourne Instruments, Natick, MA). For each mouse, we obtained LMA and Tb rhythm period (Chi-squared periodogram) and cosinor amplitude for the last 7 days in LD and the last 7 days in DD. The phase angle of entrainment (Ψ) was calculated as the difference (in hours) between the light-dark transition (ZT 12) and the acrophase of the rhythm. The acrophase of the Tb and LMA rhythms were calculated from a least squared fit during the last 7 days of LD. Chi-square periodograms were generated from the last 7 days of wheel-running data in constant darkness. The largest peak in the periodogram in the range of 12–36 h was selected as the circadian period. The amplitude of this peak was used in subsequent analysis as a proxy for the degree of circadian rhythmicity in individual animals.

### Running wheels

Mice were housed in individual cages equipped with running wheels with free access to food and water. The cages were placed in an environmental chamber (Percival Scientific, Perry, IA) maintained at 20–21 °C on a 12:12 h light:dark (LD) cycle. The environmental chamber light intensity was approximately 300 lux during L and 0 lux during D. Wheel rotations were monitored through magnetic induction, acquired continuously and stored in 5-min bins with Vitalview software (Mini-Mitter Co.). The mice were maintained in the LD cycle for at least ten days then placed into constant darkness for 21 days. Finally, a skeleton photoperiod consisting of one hour of light and 11 h of darkness was applied. Time series data were imported into Clocklab (Actimetrics) for analysis of behavioral rhythmicity. Zeitgeber time (ZT) was used to define lights-on and dark phases during the LD cycle. By convention, ZT12 was defined as lights-off. Actograms were double-plotted and the display setting was set to ‘scaled’.

### Sleep-wake monitoring and analysis

Mice were anesthetized with ketamine/xylazine [100 and 10 mg/kg respectively, IP] and then placed in a stereotaxic apparatus. Mice were implanted with four EEG screw electrodes (two frontal and two parietal electrodes; Pinnacle Technology Inc. #8403) and two flexible electromyogram (EMG) wire electrodes (Plastics One #E363/76/SPC) previously soldered to a 6-pin connector (Heilind Electronics, Inc. #853-43-006-10-001000) and the assembly was secured with dental cement^32^. The frontal electrodes were positioned 1 mm frontal and 1 mm lateral from bregma whereas the parietal electrodes were positioned 1 mm lateral from bregma and midway between bregma and lambda.

Two weeks after EEG/EMG implantation, the mice were housed individually in transparent barrels in an insulated sound-proofed recording chamber maintained at an ambient temperature of 22 ± 1 °C and on a 12 h, 12 h or 12 h light/dark cycle (lights-on at 6 A.M., Zeitgeber time: ZT0) with food and water available ad libitum. Mice were habituated to the recording cable for 5–7 days before starting polysomnographic recordings. Cortical EEG (ipsilateral frontoparietal leads) and EMG signals were amplified and digitalized with a resolution of 500 Hz using VitalRecorder software (Kissei, Japan). Sleep was recorded in baseline LD conditions followed by 5 days in constant dark.

Using SleepSign for Animal (Kissei, Japan), and with assistance of spectral analysis using fast Fourier transform (FFT), polysomnographic records were visually scored in 10 s epochs for wakefulness, NREM sleep, and REM sleep. The percentage of time spent in each condition was summarized for each group and each condition. To study sleep-wake fragmentation we analyzed the distribution of each vigilance stage in different episode lengths. Vigilance stages were separated into eight episode lengths (<30, 40–70, 80–150, 160–310, 320–630, 640–1270, 1280–2550, and >2550 s)^67,68^. For each vigilance stage, the number of episodes and the percentage of the vigilance stage occurring in each episode length bin were used to produce a time-weighted frequency histogram.

Recordings were scored again in 5 s epochs to allow for performance of an EEG power spectrum analysis. On the basis of visual and spectral analysis, epochs containing artifacts occurring during active wakefulness (with large movements) or containing two vigilance states were visually identified and omitted from the spectral analysis. Recordings containing wake artifact for more than 20% of the analysis period were removed from the spectral analysis. EEG power spectra were computed for consecutive 5 s epochs within the frequency range of 0.5–120 Hz using a FFT. The data were collapsed into 0.5 Hz bins and summed in delta, δ: 0.5–5 Hz; theta, θ: 5–9 Hz; sigma, σ: 9–15 Hz; beta, β: 15–30 Hz, low gamma, lγ: 30–60 Hz and high gamma, hγ: 60–120 Hz. For chemogenetics experiments (Fig. 6d), EEG power spectra were analyzed during the 3 h period of time post injection, starting 10 min after the injection to capture the complete range of time in which CNO affects vigilance state as previously described^32^. The data were standardized by expressing each frequency bin as a percentage relative to the same bin under baseline conditions from the same mouse and from the same time of the day (same Zeitgeber time). To analyze the FFT across the whole day in *VIP*^*cre/cre*^*::Bmal1*^*fl/fl*^ mice, *VIP*^*cre/wt*^*::Bmal1*^*fl/fl*^ mice, AVP^DTA^ mice and VIP^DTA^ mice, the FFT was analyzed in 3 h periods throughout the 24 h circadian period. The data were standardized by expressing each frequency band as a percentage relative to the 24 h frequency band power.

### In vivo fiber photometry

A total of 12 heterozygous *VIP-ires-Cre* mice were used for photometry experiments. Only mice bearing accurate targeting of the photometry fiber and injection sites are included in the results, hence the final cohort size of 4.

Surgeries: Two weeks after the viral vector injection of AAV10-DIO-EF1ɑ-GCaMP6s, the mouse was implanted with a custom-made photometry fiber and EEG/EMG headset. The photometry fibers were manufactured in-house and comprised a fiber optic cable (400 µm outer diameter, ThorLabs FP400URT) inserted into an aluminum ferrule (400 µm internal diameter, ThorLabs SFLC440-10), epoxied into place (PFP 353ND, Precision Fiber Products), cleaved to size and polished. The EEG headset was also manufactured in-house (as described above), with the exception that the left frontal electrode screw was removed from the assembly to allow photometry fiber implantation. The mouse was prepared for stereotaxic surgery as described above (Stereotaxic brain injections). Once secured in the stereotaxic frame, the EEG headset was implanted as described above (Sleep-wake monitoring and analysis). The burr hole over the SCN was reopened and the photometry fiber was implanted immediately dorsal to the SCN (0.1 mm dorsal to the injection site). The whole assembly was affixed to the skull using a mixture of cyanoacrylate glue and dental cement. An additional layer of dental cement was then applied to insulate the headset and provide structural stability. The mouse was then allowed to recover for 2 weeks prior to recordings.

Photometry behavioral recordings: Recordings were carried out in a sound-proofed and light-tight chamber, maintained at a constant temperature and humidity. Prior to the experimental recordings, mice were kept on a 12:12 light-dark schedule. Clear, plexiglass barrels were used as the animal’s home cage and food and water were available ad libitum. Mice were allowed to habituate to the recording chambers, EEG/EMG cable and photometry patch cable for at least 3 days prior to behavioral recordings before being placed in DD at lights off on the last day of habituation. Simultaneous EEG/EMG and in vivo fiber photometry recordings were then made at CT2-5 in the first cycle after the onset of DD and at CT 12–16 (24 h after the onset of DD). A 20-min light pulse was administered at CT 14 (26 h after the onset of DD).

Simultaneous EEG/EMG and photometry recording setup and analyses: For EEG/EMG recordings, the EEG/EMG headset was connected, via a low weight custom-made cable, to a freely moving electrical commutator. The signal from the commutator was directed to an AM Systems Model 1700 amplifier (amplifed ×10 K gain) and digitized at 500 Hz using a Micro-1401-3 (Cambridge Electronic Design).

To simultaneously record the GCaMP6s signal, light from a 465 nm blue LED (the excitation wavelength for GCaMP6s, LEDC1-B_FC Doric) and a 405 nm UV LED (LEDC1-405_FC) was directed to a fiber optic mini-cube containing UV and GFP filters (FMC5_AE(405)_AF(420-450)_E1(460-490)_F1(500-550)_S, Doric) via a 200 µm, 0.22 NA fiber patch cable. A sample patch cord (400 µm, 0.48 nm) was mated to the photometry fiber ferrule on the mouse’s head to deliver blue and UV light to the SCN and collect the emitted fluorescence from GCaMP6s-expressing cells. This emitted signal was directed back through the filter cube to two photodetectors (Newport 2151) via 600 µm, 0.66 NA fiber patch cables. The signal was amplified at the photodetector (DC gain: 2 × 10^10^) and digitized at 500 Hz (Micro-1401-3, Cambridge Electronic Design). Recordings in which UV excitation of the tissue resulted in a moving signal that ran parallel to the recorded calcium-dependent GCaMP6s signal were assumed to be contaminated with movement artifact and discarded from the analysis. Data were acquired using Spike 2.13 software and sleep-wake states were analyzed offline. For automatic data scoring, EMG root mean square, EEG delta power and EEG theta:delta ratio were used to distinguish between sleep states. All sleep records were then manually scored by an investigator blinded to the experimental conditions.

After behavioral sleep stage scoring, the GCaMP6s signal and sleep staging timestamps were exported to MATLAB for further analysis. Data were downsampled to 1 Hz and fitted to a second order exponential curve to correct for bleaching. This curve was designated as F0 and used to calculate δF/F = F_t_-F0/F0, where F_t_ denotes the GCaMP6s signal at time *t*. To normalize fluorescence levels between mice, δF/F was then processed as a z-score. At transitions, the GCaMP6s signal was zeroed by calculating the median value of the 60 s preceding the transition and subtracting this from the GCaMP6s signal throughout the transition (i.e., 60 s before to 60 s after the transition occurred).

### Rabies tracing

For monosynaptic retrograde modified rabies tracing we first made injections of a mixture of 60 nl AAV8- EF1ɑ -DIO-TVA and 60 nl AAV8-CAG-DIO-RG into the SCN as described above (Stereotaxic brain injections). Four weeks after the initial surgeries, 200 nl of EnvA-ΔG-eGFP was injected into the same place. Mice were perfused 11 days following the second stereotaxic injection and processed for histology (as described in Immunohistochemistry). In brief, one entire series was immunolabelled for dsRed to allow visualization of TVA-mCherry and then mounted. Neurons containing both TVA-mCherry and eGFP were considered the starter population. Each eGFP positive neuron in the brain was counted and registered to the mouse atlas of Paxinos and Franklin^66^. The profile of afferent inputs is presented as the number of eGFP expressing neurons within a particular brain region as a percent of the total number of eGFP neurons counted in the whole brain (the input fraction).

### ChR2-assisted circuit mapping (CRACM)

For CRACM experiments, we injected *VIP-IRES-Cre* mice (*n* = 5) with of AAV-DIO-ChR2-mCherry unilaterally into the SCN and placed a second injection of of Fluorescent-conjugated Cholera Toxin Subunit B (F-CTB; green Alexa Fluor-488 conjugated-CTB; Invitrogen) into the ipsilateral DMH of the same mice, to retrogradely label SPZ neurons projecting to the DMH.

Five to six weeks after the AAV and F-CTB injections, mice were sacrificed for in vitro electrophysiological recordings. Mice were deeply anaesthetized with isoflurane (5% in oxygen) via inhalation and transcardially perfused with ice-cold cutting ACSF (N-methyl-D-glucamine, NMDG-based solution) containing (in mM): 100 NMDG, 2.5 KCl, 1.24 NaH_2_PO_4_, 30 NaHCO_3_, 25 glucose, 20 HEPES, 2 thiourea, 5 Na-L-ascorbate, 3 Na-pyruvate, 0.5 CaCl_2_, 10 MgSO_4_ (pH 7.3 with HCl when carbogenated with 95% O_2_ and 5% CO_2_; 310–320 mOsm). Mouse brains were then quickly removed and sectioned in coronal slices (250 µm thickness) in ice-cold NMDG-based ACSF using a vibrating microtome (VT1200S, Leica). We first incubated the slices containing the hypothalamus for 5 min at 37 °C, then we transferred them into a holding chamber at 37 °C containing normal ACSF (Na-based solution) for 10 min. Following this two-step incubation we let the brain slices gradually return to room temperature (~1 h) before transferring them to the recording chamber where they were submerged and perfused (1.5 ml/min) with Na-based ACSF. Normal ACSF (Na-based solution) contained (in mM): 120 NaCl, 2.5 KCl, 1.3 MgCl_2_, 10 glucose, 26 NaHCO_3_, 1.24 NaH_2_PO_4_, 4 CaCl_2_, 2 thiourea, 1 Na-L-ascorbate, 3 Na-pyruvate (pH 7.3–7.4 when carbogenated with 95% O_2_ and 5% CO_2_; 310–320 mOsm). We recorded SPZ neurons (retrogradely labeled with F-CTB) using a combination of fluorescence and infrared differential interference contrast (IR-DIC) microscopy. We used a fixed stage upright microscope (BX51WI, Olympus America Inc.) equipped with a Nomarski water immersion lens (Olympus 40×/0.8 NAW) and IR-sensitive CCD camera (ORCA-ER, Hamamatsu, Bridgewater, NJ, USA) and we acquired real time images using MATLAB (MathWorks) script software. We recorded SPZ neurons in whole-cell and cell-attached configurations using a Multiclamp 700B amplifier (Molecular Devices, Foster City, CA, USA), a Digidata 1322A interface, and Clampex 9.0 software (Molecular Devices). Neurons showing changes in input resistance of greater than 10% over the duration of the recording were excluded from the analysis. For all recordings, we recorded at a holding potential of 0 mV and using a Cs-methane-sulfonate-based pipette solution. The Cs-methane-sulfonate-based pipette solution contained (in mM): 125 Cs-methane-sulfonate, 11 KCl, 10 HEPES, 0.1 CaCl_2_, 1 EGTA, 5 Mg-ATP and 0.3 Na-GTP (pH adjusted to 7.2 with CsOH, 280 mOsm). For all the recordings, we added 0.5% biocytin in the pipette solutions to mark the recorded neurons. We photostimulated SCN^VIP^ axon terminals in the SPZ using a full-field (~10 mW/mm^2^, 1 mm beam width) blue light-emitting diode (470 nm wavelength; #M470L2-C4; Thorlabs) coupled to the epifluorescence pathway of the microscope. We first tested the effects of photostimulation on the action potential firing of SPZ neurons in cell-attached mode, using train stimulations (train-duration: 10 s, frequency: 10 Hz, light pulse duration: 10 ms). We then switched to whole-cell mode (and ACSF containing 1 mM of kynurenic acid). To record IPSCs, we applied 10 ms light pulses (0.1 Hz, for a minimum of 30 trials) to the brain slice in order to elicit photo-evoked IPSCs. At the end of the recordings, recorded slices and adjacent slices containing the AAV and F-CTB injection sites were fixed overnight in 10% buffered formalin for further histological processing.

### CRACM Data analysis

Recording data were analyzed using Clampfit 10 (Molecular Devices), MiniAnalysis 6 software (Synaptosoft) and MatLab (MathWorks) software. Figures were generated using Igor Pro version 6 (WaveMetrics), Prism 7 (GraphPad) and Photoshop (Adobe) software. To ensure unbiased detection of the synaptic events, the IPSCs were detected and analyzed automatically using MiniAnalysis. We considered SPZ neurons to be responsive to photostimulation if their IPSC probability during the first 50 ms following the light pulses was greater than 50% (baseline IPSC probability = 21.08 ± 3.15%, *n* = 20)^69^. We calculated the latency of the photo-evoked IPSCs as the time difference between the start of the light pulse and the 5% rise point of the first IPSC^70^.

### Immunohistochemistry

Mice were sacrificed by deep anesthesia with 200 mg/kg of chloral hydrate, and transcardially perfused with 20 ml saline, followed by 100 ml of neutral phosphate buffered formalin (4%, Fischer Scientific Co.). Brains were removed, incubated in 20% sucrose at 4 °C until they sank, and then sectioned at 40 µm on a freezing microtome in three series. Sections were washed in phosphate buffered saline (PBS), and incubated in primary antiserum (dsRed rabbit polyclonal antibody (1:10 K; Clontech; catalog number 632496), AVP antibody(1:8K; Peninsula Lab, LLC catalog number T-5048​), VIP antibody (1:10K; Millipore, catalog number AB983), or hrGFP rabbit polyclonal antibody (1:10 K; Vitality; 240142-51) diluted in PBS containing 0.3% Triton X-100 (PBST) and 0.2% sodium azide for one day at room temperature. For fluorescent detection of primary antibodies, sections were washed three times in PBS and incubated in fluorescent secondary antiserum for 2 h (against appropriate species IgG, 1:500, Jackson ImmunoResearch). Sections were then washed twice in PBS before being mounted on positive charged slides (Denville Scientific, Inc) and imaged on an Olympus BX61VS slide scanner or Zeiss LSM 5 Pascal confocal microscope. For mice injected used for retrograde rabies tracing experiments, the retinas were removed following perfusion and incubated in 20% sucrose at 4 °C until they sank. The retina was sliced rostral-caudally in 10 µm slices on a cryostat and retinal sections were collected directly onto positively charged slides (Denville Scientific, Inc) that had been pre-subbed in 1% gelatin solution, before imaging native eGFP.

For brain slices used for CRACM experiments, we further processed the recorded SCN-SPZ containing slices over-night with streptavidin-conjugated Alexa Fluor-405 (blue; 1: 500; Invitrogen)^21^ to label the recorded SPZ neurons filled with biocytin. We then wet mounted brain slices containing the SCN and DMH and imaged them using a Zeiss LSM 5 Pascal confocal microscope and Zen 2009 software (Zeiss), and mapped the distribution of the SPZ recorded neurons onto a template image. We also projected the F-CTB injection sites from each mouse onto a series of standardized sections^66^ and compiled these using a custom-made Python script (www.python.org)^71^ in order to construct a heatmap.

### sNuc-Seq

*sNuc-Seq Data Generation:* Six-week old (juvenile; *n* = 2) and 10-week old (adult, *n* = 1) *VIP-IRES-Cre::H2b-TRAP* male siblings were bred from the same *VIP-IRES-Cre x H2b-TRAP* mouse line. One hour after light cycle onset, the *VIP-IRES-Cre::H2b-TRAP* mice were removed from their home cage and rapidly decapitated in order to minimize stress-related changes in gene expression. Brains were extracted quickly, chilled briefly in ice-cold DMEM/F12 media, and then sectioned coronally at 1-mm intervals in a chilled stainless steel brain matrix. The brain sections containing the SCN and sections adjacent to the SCN were collected in ice-cold RNAlater (Qiagen catalog # 76106). Each section was imaged using a fluorescence stereoscope (Zeiss Discovery V8) in order to visualize fluorescently labeled cells in the SCN for dissection. Dissected tissue was pooled across mice by age group (juvenile, adult) and placed into ice-cold RNAlater overnight in the dark at 4 °C. The following morning, the RNAlater-preserved tissue samples were processed into single-nuclei suspensions according to the sNuc-Seq method of Habib et al.^42^. In brief, the two SCN tissue samples (juvenile, adult) were Dounce homogenized (Wheaton tissue grinders, catalog # 357538) and separated by density gradient centrifugation as described in the published protocol^42^. Each pellet was then re-suspended in 0.5 mL sNuc-Seq resuspension buffer^42^ and filtered through a 20 μm mesh (Miltenyi Biotec pre-separation filters, catalog # 130-101-812) to remove clumped nuclei and larger debris. Nuclei were counterstained with two drops of NucBlue Live ReadyProbes Reagent (ThermoFisher Scientific catalog # R37605) per mL of nuclei. Nuclei were sorted by MoFlo Astrios EQ cell sorter (Harvard Bauer core facility), gating for NucBlue + /mCherry+ singlets and distributing them individually to wells of 96-well plates containing 5 μL per well of nuclei capture buffer (1% beta-mercaptoethanol in Qiagen Buffer TCL; catalog # 1031576). Each plate was loaded with equal numbers of samples from juvenile and adult SCN tissue. Loaded plates were centrifuged for 5 min at 2500 × *g* and 4 °C to ensure nuclei reached the capture buffer and frozen at −80 °C for up to 4 weeks until use. Sample plates were transferred to an RNA-dedicated PCR cabinet, thawed quickly, and immediately purified with RNA-Clean XP (Beckman Coulter, catalog # A63987; 2.5:1 ratio of RNA-Clean XP to sample volume), eluting in 4 μL Smart-seq2 buffer (2 μL Smart-seq2 cell lysis buffer, 1 μL 100 μM oligo-dT primer, and 1 μL 10 mM dNTP mix)^72^. cDNA was then generated and amplified as described in the Smart-Seq2 protocol^72^, except that 22 PCR cycles were used for the amplification step. After the PCR step, 10 μL of each sample was transferred to new 96-well plates for subsequent processing. Amplified cDNA was purified with two rounds of SPRIselect (Beckman Coulter, catalog # B23318; 0.8:1 ratio of SPRIselect to sample volume), eluting in ultrapure water. cDNA concentration was measured by Qubit fluorometry in a microplate format, each well containing 2 μL of cDNA sample and 98 μL of Qubit working solution (ThermoFisher Scientific, catalog # Q32854). Qubit readings were collected with a fluorescence microplate reader (Molecular Devices SpectraMax M5; Harvard Medical School ICCB-Longwood core facility) and used to calculate DNA concentrations by comparing each sample’s Qubit reading to standards in a 50% dilution series (*n* = 8 standards; range, 0.25–0.01 ng/μL plus a negative control, 0.0 ng/μL). cDNA samples were then diluted to 0.15 ng/μL with ultrapure water and processed into sequencing libraries with sample-specific indices using the Nextera DNA Sample Prep Kit and Nextera XT Index Kit v2 sets A–D (Illumina, catalog #s FC-131-1096, FC-131-2001, FC-131-2002, FC-131-2003, FC-131-2004, respectively). Nextera XT was used at ¼ reaction volumes but otherwise according to manufacturer’s instructions. Sequencing libraries were purified with two rounds of SPRIselect (0.8:1 ratio of SPRIselect to sample volume) and eluted in a single volume of ultrapure water. Library pool concentration was measured by Qubit according to the manufacturer’s instructions, sized using Agilent BioAnalyzer high sensitivity kit, and then diluted with ultrapure water to a size-corrected concentration of 2 nM. The diluted library pool was denatured and further diluted to 2pM loading concentration according to Illumina NextSeq recommendations, then sequenced with a NextSeq 500 v2 75cycle sequencing kit (Illumina, catalog # FC-404-2005).

*sNuc-Seq data processing*. Base calls and demultiplexing were performed with bcl2fastq2 v2.20.0. Reads were aligned to the mouse genome (build: mm10, GRCm38) by HISAT2 v2.1.0 (using “-k 2”)^73^. Aligned reads were converted to BAM using SAMtools v1.8.0^74^. Duplicate and low-quality reads were removed with Picard 2.17.0 (http://broadinstitute.github.io/picard). Reads were assigned to features (genes) using the feature Counts from the Subread package v1.6.0 (using recommended setting and a custom meta-annotation that merges overlapping isoforms)^75^. Expression values were calculated by counting the reads assigned to each gene.

*sNuc-Seq data analysis*. Clustering and cluster marker analyses were performed in Seurat v2.3.4^76^ using the standard workflow and default settings except where indicated. First, the gene expression values were imported and log-normalized. Highly variable genes (*n* = 943) were selected with Seurat’s FindVariableGenes function for having higher ratios of dispersion relative to mean expression. These highly variable genes were used for principal component analysis (PCA) with Seurat’s RunPCA function. Transcriptomes were then clustered across the first 10 principal components (PCs) using Seurat’s FindClusters function, resolution set to 1. Clusters were numbered based on their position in hierarchical clustering dendrogram. To identify cluster markers, differential expression analysis was performed with Seurat’s FindAllMarkers function under default settings. Clusters were assigned as “SCN” or “non-SCN” based on the anatomical expression patterns of top cluster markers observed in the Allen Mouse Brain Atlas^77^ (*Lhx1*; *Vipr2*; *Reln*; *Ecel1*). The SCN clusters were re-clustered by excluding the non-SCN cluster and then repeating the workflow described above, beginning with the variable gene selection (689 variable genes used for PCA, 12 PCs used for clustering).

Plots were generated with Seurat v2.3.4 and pheatmap ﻿v1.0.12 packages for R.

### VIP RNAScope^TM^ in situ hybridization

RNAScope^TM^ in situ hybridization was performed to label VIP mRNA in mouse brain tissue. For this, 30 μm SCN brain sections were cut on a freezing microtome and mounted on Superfrost Plus slides in RNAse free conditions. RNAscope hybridization was performed on these sections using the RNAScope Multiplex Flourescent Reagent Kit V2 (Catalog # 323100, Advanced Cell Diagnostics). The sections were then pretreated with hydrogen peroxide for 20 min in room temperature and target retrieval was performed by placing the slides in a steamer at 99 °C for 5 min. Next, sections were dehydrated in 100% alcohol and air-dried for 5 min. Sections were then treated with protease reagent (Protease III, RNAscope) for 30 min at 40 °C. After rinsing in sterile water, sections were incubated in RNAscope probe for VIP-C1 (RNAscope^®^ Probe- Mm-Vip;Cat No. 415961;Advanced Cell Diagnostics) for 2 h at 40 °C for the hybridization reaction to occur. Sections were then subjected to three amplification steps at 40 °C (AMP1-FL and AMP2-FL: 30 min each; AMP3-FL: 15 min) followed by incubation in HRP –C1 for 15 min. Sections were then incubated in TSA plus Fluorescein fluorophore (Catalog # NEL741001, Perkin Elmer) for 30 min at 40 °C to enable visualization of VIP mRNA in green. Finally, slides were incubated in HRP blocker for 15 min at 40 °C before being dried overnight and cover-slipped using Vectashield mounting medium (Catalog # H-1400, Vector laboratories).

### Statistics

All statistics were carried out using Prism v6 or v7 (GraphPad software) unless otherwise indicated. For our circadian variables in our crossed mice (Fig. 1), one-way ANOVAs were used to determine group differences across phenotypes in these values, with Sidak tests serving as *post hoc* analyses. For our cell-specific ablations experiments, unpaired *t* tests were used to determine differences between *VIP*^*DTA*^ and *AVP*^*DTA*^ mice (Fig. 3). For sleep-wake analyses (Figs. 2, 4, 6, S5, S6, S7, S8): following confirmation that the data met the assumptions of the ANOVA model, two-way ANOVA followed by a post hoc Bonferroni test were used to compare the effects of genotype or drug injection and time period on sleep-wake parameters, the effect of genotype or drug injection and the distribution of vigilance episodes, or the effect of genotype or drug injection and cortical EEG power density. The individuals performing the sleep-wake analysis were blinded to the initial immunohistochemical assessment of the injection sites and to the mouse genotype.

For photometry experiments (Fig. 7 and S9): to quantify the effect of the light pulse or of a state transition upon GCaMP6s activity, the mean GCaMP6s signal was averaged during the 10 seconds preceding the light pulse or the state transition and compared with GCaMP6s signal averaged 10 s afterwards. Statistical analyses were carried out using paired *t*-tests. To quantify GCaMP6s activity during different states and/or at different times, the GCaMP6s signal was averaged over the duration of the state and compared using repeated measures one-way ANOVA or repeated measures two-way ANOVA, utilizing a post hoc Sidak test where applicable.

For retrograde rabies tracing (Fig. 9), we repeated this experiment on *n* = 3 different mice. In two of the mice, there was some leakage of the starter population into the rostrally adjacent VMPO, therefore we only quantified % inputs to SCN neurons for *n* = 1 mouse.

Sample size and power calculations were performed *post hoc* at http://www.biomath.info, using means and standard deviations derived from our analysis. The present study was sufficiently powered to detect effect sizes.

## Supplementary information

Description of Additional Supplementary Files 

Supplementary Information

## Data Availability

The data that support the findings of this study are presented within this paper or its supplementary materials or are available from the corresponding author upon reasonable request. The raw data files (FASTQ) and processed expression values reported in this study were deposited in the Gene Expression Omnibus database under GSE150770.

## Code availability

All custom code used in this manuscript has been previously published, and is available in the relevant cited publications.
