# Supplementary material for: Suprachiasmatic VIP neurons are required for normal circadian rhythmicity and comprised of molecularly distinct subpopulations
Source: Nat Commun. 2020 Sep 2;11:4410. doi: 10.1038/s41467-020-17197-2 (PMC7468160; doi:10.1038/s41467-020-17197-2)
Supplement: Supplementary file 1 — Description of Additional Supplementary Files [file 41467_2020_17197_MOESM1_ESM.pdf]

Anatomical Abbreviations and Quantification for Figure 9

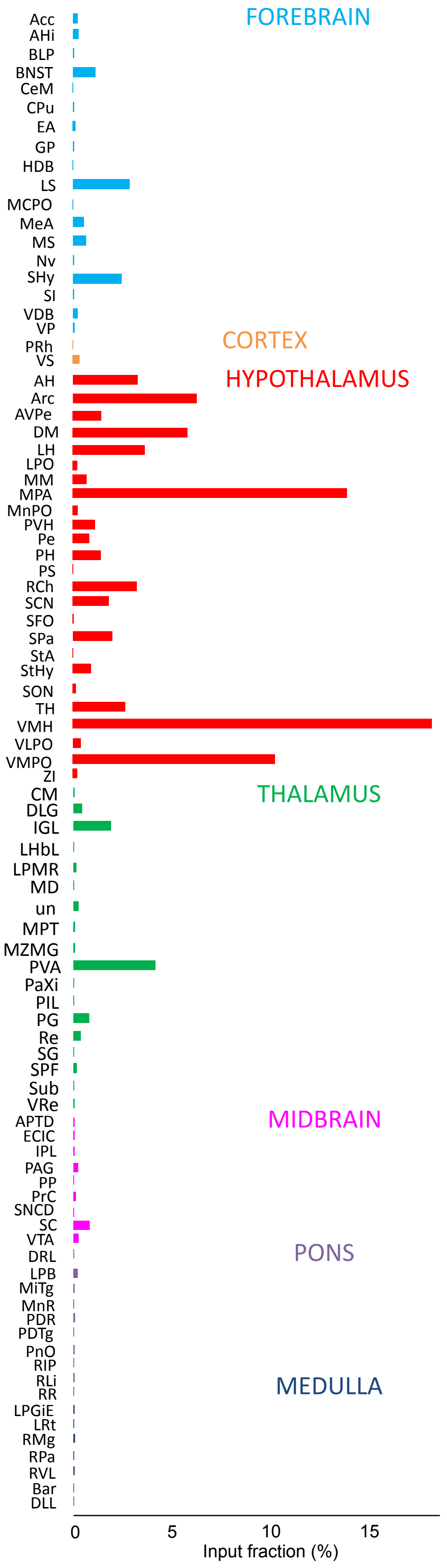

Abbreviation List: 3V, 3rd ventricle; ac, anterior commissure; Acc, accumbens nucleus; AH, anterior hypothalamic area, anterior part; Ahi, amygdalohippocampal area; alv, alveus of the hippocampus; APTD, anterior pretectal nucleus, dorsal part; Arc, arcuate hypothalamic nucleus; AVPe, anteroventral periventricular nucleus; Bar, Barrington's nucleus; BLP, basolateral amygdaloid nucleus, posterior part; BNST, bed nucleus of the stria terminalis; CeM, central amygdaloid nucleus, medial division; CM, central medial thalamic nucleus; CPu, caudate putamen (striatum); DLG, dorsal lateral geniculate nucleus; DLL, dorsal nucleus of the lateral lemniscus; DM, dorsomedial hypothalamic nucleus; DRL, dorsal raphe nucleus, lateral part; EA, extended amygdala; ECIC, external cortex of the inferior colliculus; fx, fornix; GP, globus pallidus; HDB, nucleus of the horizontal limb of the diagonal band; IGL, intergeniculate leaflet; IPL, interpeduncular nucleus, lateral subnucleus; LH, lateral hypothalamic area; LHbL, lateral habenular nucleus, lateral part; LPB, lateral parabrachial nucleus; LPGiE, lateral paragigantocellular nucleus, external part; LPMR, lateral posterior thalamic nucleus, mediorostral part; LPO, lateral preoptic area; LRt, lateral reticular nucleus; LSD, lateral septal nucleus, dorsal part; LSV, lateral septal nucleus, ventral part; LV, lateral ventricle; MCPO, magnocellular preoptic nucleus; MD, mediodorsal thalamic nucleus; MeA, medial amygdaloid nucleus, anterior part; MiTg, microcellular tegmental nucleus; MM, medial mammillary nucleus, medial part; MnPO, median preoptic nucleus; MnR, median raphe nucleus; MPA, medial preoptic area; MPT, medial pretectal nucleus; MS, medial septal nucleus; MZMG, marginal zone of the medial geniculate; Nv, navicular postolfactory nucleus; opt, optic tract; ox, optic chiasm; PAG, periaqueductal gray; PaXi, paraxiphoid nucleus of thalamus; PDR, posterodorsal raphe nucleus; PDTg, posterodorsal tegmental nucleus; Pe, periventricular hypothalamic nucleus; PG, pregeniculate nucleus; PGMC, pregeniculate nucleus, magnocellular part; PGPC, pregeniculate nucleus, parvicellular part; PH, posterior hypothalamic nucleus; PIL, posterior intralaminar thalamic nucleus; PnO, pontine reticular nucleus, oral part; PP, peripeduncular nucleus; PrC, precommissural nucleus; PRh, perirhinal cortex; PS, parastrial nucleus; PVA,

paraventricular thalamic nucleus, anterior part; PVH, paraventricular hypothalamic nucleus; Re, reuniens thalamic nucleus; RIP, raphe interpositus nucleus; RLi, rostral linear nucleus (midbrain); RMg, raphe magnus nucleus; RR, retrorubral nucleus; RVL, rostroventrolateral reticular nucleus; SC, superior colliculus; SCN, suprachiasmatic nucleus; SFO, subfornical organ; SG, suprageniculate thalamic nucleus; Shy, septohypothalamic nucleus; SHy, septohypothalamic nucleus; SI substantia innominate; SNCD, substantia nigra, compact part, dorsal tier; SON, supraoptic nucleus; Spa, subparaventricular zone of the hypothalamus; SPF, subparafascicular thalamic nucleus; StA, strial part of the preoptic area; STLI, bed nucleus of the stria terminalis, lateral division; STLP, bed nucleus of the stria terminalis, lateral division, posterior part; STLV, bed nucleus of the stria terminalis, lateral division, ventral part; STMA, bed nucleus of the stria terminalis, medial division, anterior part; STMAL, bed nucleus of the stria terminalis, medial division, anterolateral part; Sub, submedius thalamic nucleus; Te, terete hypothalamic nucleus; un, unassigned thalamic area, located between the fasciculus retroflexus; VDB, nucleus of the vertical limb of the diagonal band; VLPO, ventrolateral preoptic nucleus; VMH, ventromedial hypothalamic nucleus; VMPO, ventromedial preoptic nucleus; VP, ventral pallidum; VRe,ventral reuniens thalamic nucleus; VS, ventral subiculum; VTA, ventral tegmental area, ZI, zona incerta.
