# Supplementary material for: Suprachiasmatic VIP neurons are required for normal circadian rhythmicity and comprised of molecularly distinct subpopulations
Source: Nat Commun. 2020 Sep 2;11:4410. doi: 10.1038/s41467-020-17197-2 (PMC7468160; doi:10.1038/s41467-020-17197-2)
Supplement: Supplementary file 2 — Supplementary Information [file 41467_2020_17197_MOESM2_ESM.pdf]

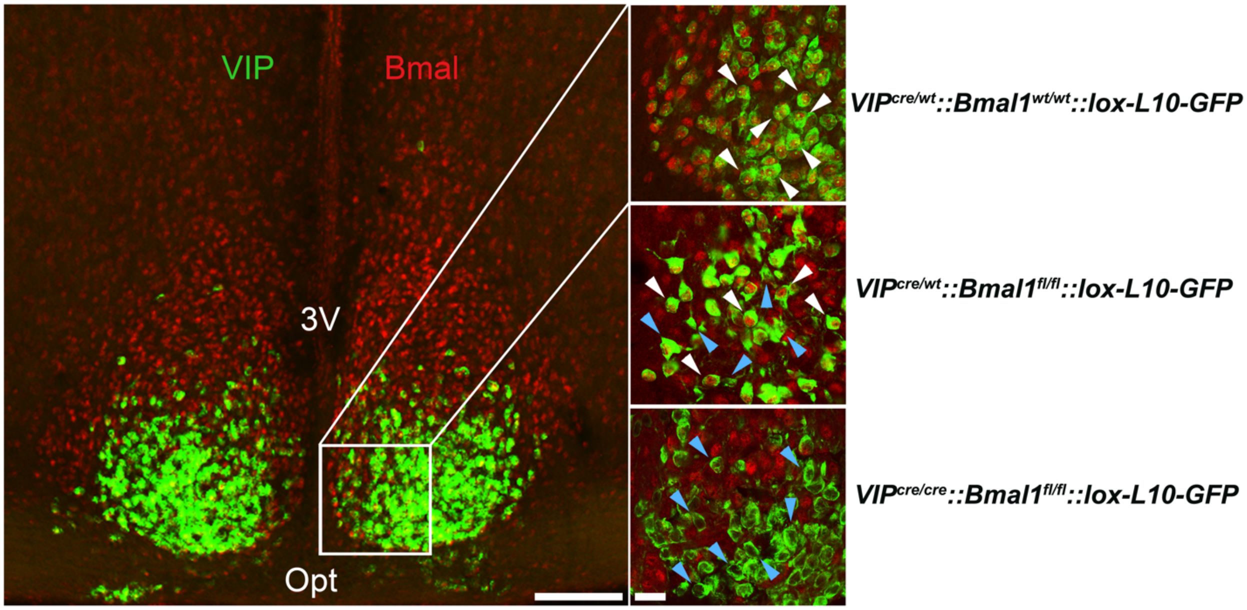

Figure S1. Deletion of *Bmal1* from SCN^{VIP} neurons via genetic cross.

To quantify the efficiency and specificity of *Bmal1* deletion in *VIP$^{cre/cre}$::Bmal$^{fl/fl}$* mice we generated triple cross *VIP-ires-Cre::Bmal1$^{fl/fl}$::R26-loxSTOPlox-L10-GFP* mice. Example confocal images of the SCN of *VIP$^{cre/wt}$::Bmal1$^{wt/wt}$::R26-loxSTOPlox-L10-GFP* (L10-GFP) mice showing 100% co-localization between VIP (L10-GFP, green) and *Bmal1* (red; left and top right). In *VIP$^{cre/wt}$::Bmal1$^{fl/fl}$::L10-GFP* mice, ~ 70% of VIP-positive neurons continued to express *Bmal1* (center right). In *VIP$^{cre/cre}$::Bmal1$^{fl/fl}$::L10-GFP* mice, 0% of VIP-positive neuron expresses *Bmal1* (bottom right), although *Bmal1* continued to be abundantly expressed in adjacent non-VIPergic neurons of the ventral SCN. White arrows indicate VIP-*Bmal1* co-localization, while blue arrows show absence of co-localization. Scale bars: left, 100 μm and right, 20 μm.

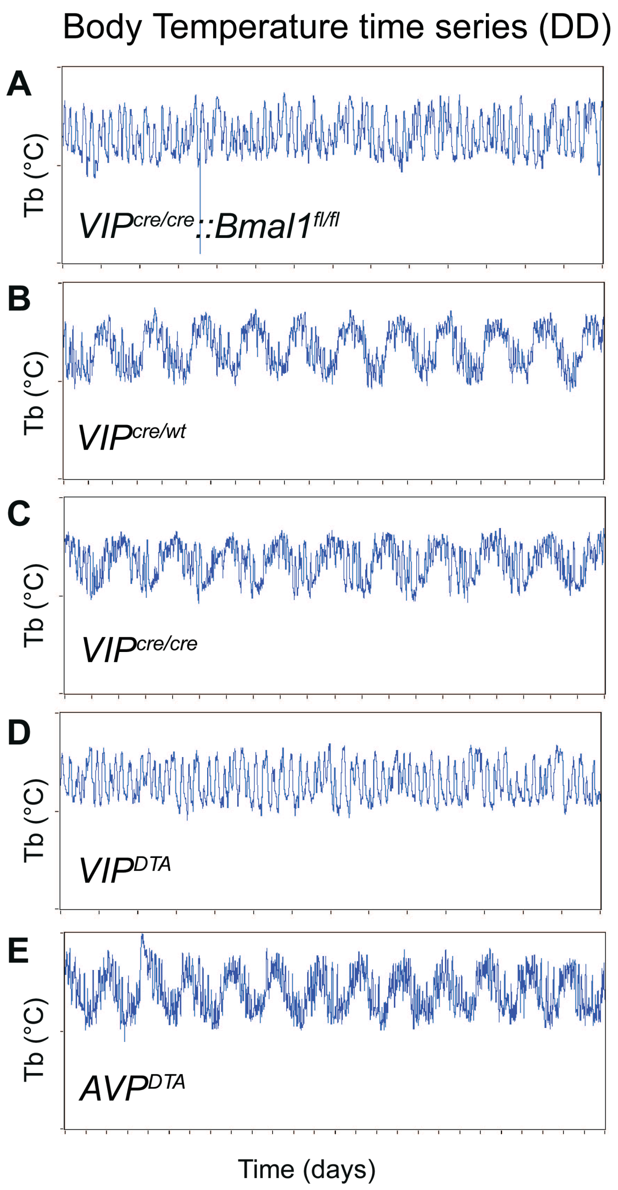

Figure S2. Tb rhythm waveforms in DD in *VIP$^{cre/wt}$*, *VIP$^{cre/cre}$*, *VIP$^{cre/cre}$::Bmal1$^{fl/fl}$*, VIP^{DTA}*, and* AVP^{DTA} *mice*. Related to Figure 1 and 3. Selective deletion of *Bmal1* in SCN^{VIP} neurons (A) has clear visible disruptive effective on the Tb waveform compared to *VIP$^{cre/wt}$ (B)* and *VIP$^{cre/cre}$* (C) mice, yet these rhythms, per periodogram analysis, retain a circadian component in the *VIP$^{cre/cre}$::Bmal1$^{fl/fl}$* mouse. Selective ablation of SCN^{VIP} neurons (D), but not SCN^{AVP} neurons (E), severely disrupts circadian rhythms of Tb.

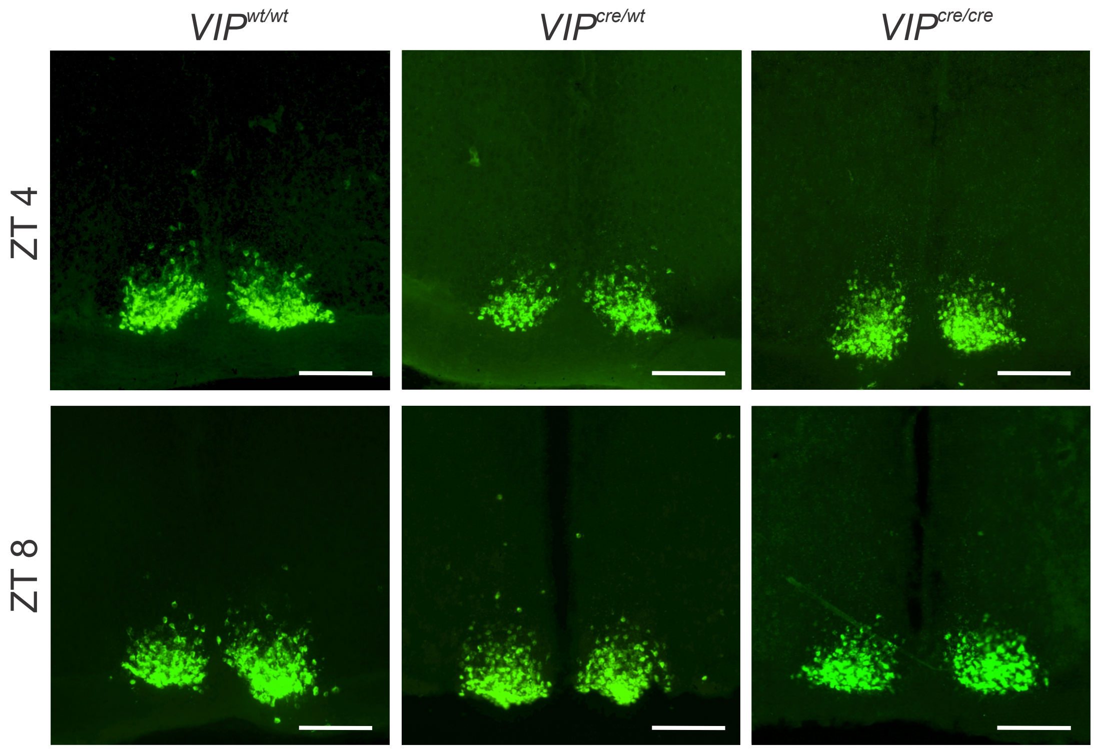

Figure S3. VIP mRNA expression in *VIP*$^{wt/wt}$, *VIP*$^{cre/wt}$ and *VIP*$^{cre/cre}$ conditions. Related to Figure 1. RNAscope-based *in situ* hybridization for *VIP* mRNA within the SCN is shown from brains sacrificed at ZT4 (upper panels) and ZT8 (lower panels) for *VIP*$^{wt/wt}$, *VIP*$^{cre/wt}$ and *VIP*$^{cre/cre}$. VIP transcript levels are visually comparable across the wild type, heterozygous and homozygous condition in *VIP-ires-Cre* mice. Scale bars: 100 μm.

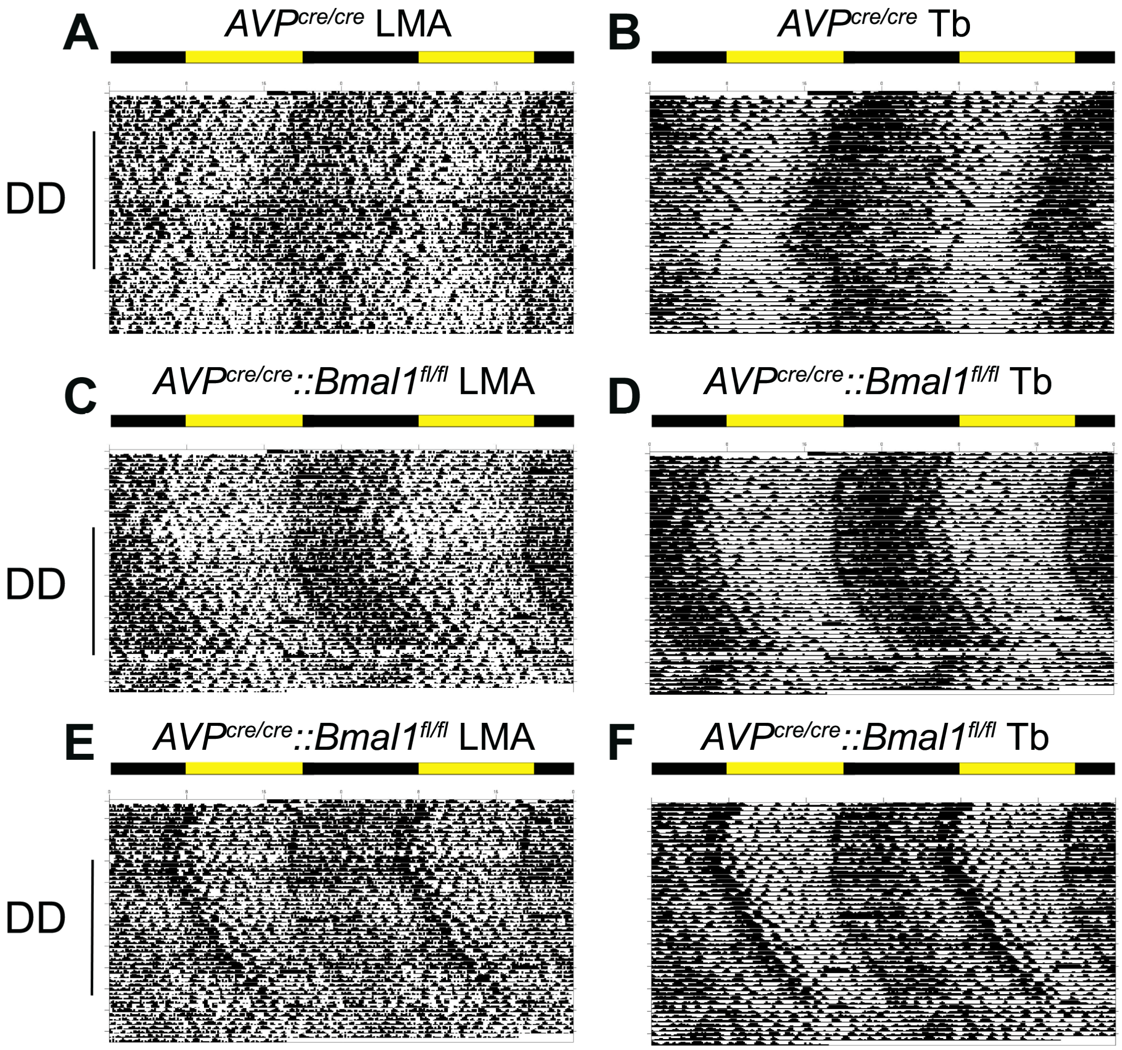

Figure S4. Loss of the molecular clock in SCN^{AVP} neurons on LMA and Tb rhythms. Related to Figure 1. A-B. Example actogram (LMA; A,C,E) and Tb variation (B,D,F; grey scale: darker represents higher temperature) for an *$AVP^{cre/cre}$* mouse (A-B) and two *$AVP^{cre/cre}$::$Bmal1^{fl/fl}$* mice (C-F) during at least 10 days in light/dark condition (LD), followed by at least 3 weeks days in constant dark (DD), followed by at least seven days of LD. Note that *$AVP^{cre/cre}$::$Bmal1^{fl/fl}$* mice retain coherent rhythms of LMA and Tb, but exhibit a lengthened period compared to *$AVP^{cre/cre}$* mice.

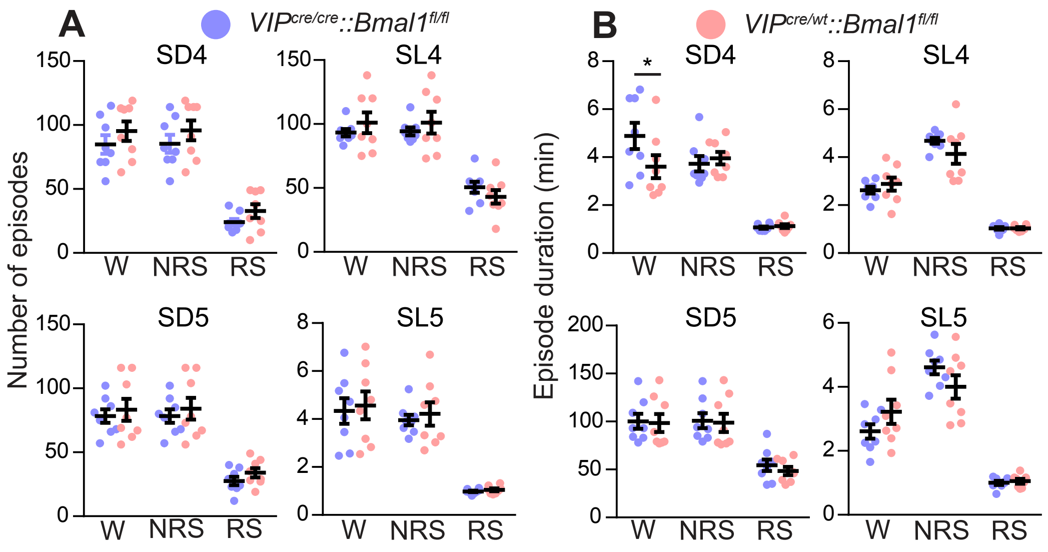

Figure S5. Sleep-wake qualitative changes following loss of the molecular clock in SCN^VIP neurons. Related to Figure 2. **(A)** Number of episodes (mean ± s.e.m.) of wake (W), NREM sleep (NRS) or REM sleep (RS) and **(B)** episode duration (mean ± s.e.m.) of W, NRS or RS during the 4th and 5th subjective dark (SD4, SD5), and 4th and 5th subjective light (SL4, SL5) periods in *VIP$^{cre/cre}$::Bmal1$^{fl/fl}$* (blue, n = 8) and *VIP$^{cre/wt}$::Bmal1$^{fl/fl}$* (red, n = 8) mice. * $p < 0.05$, two-way ANOVA followed by a *post hoc* Bonferroni test.

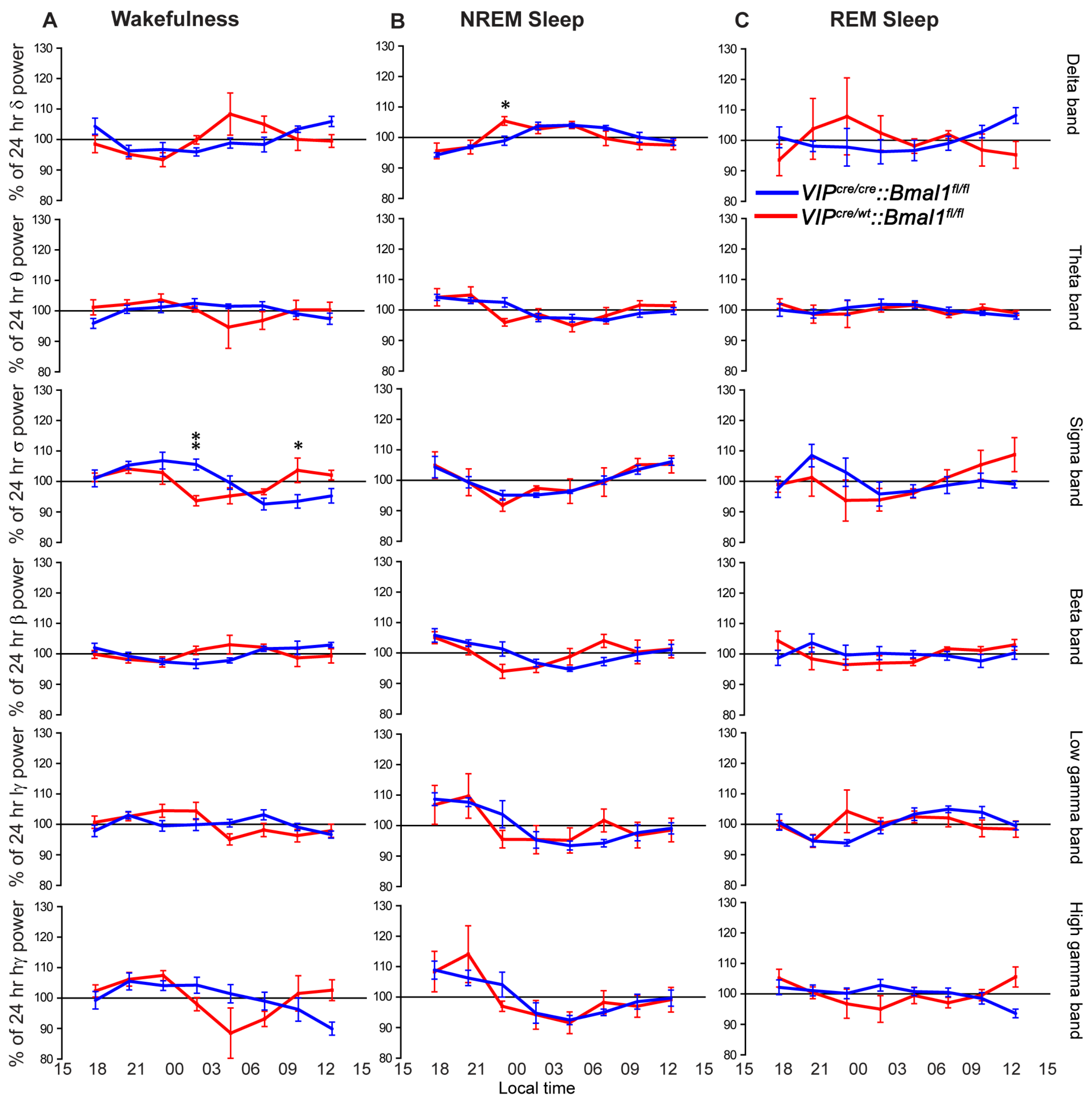

Figure S6. Sleep-wake cortical EEG power band activity across the 24 hr period following loss of the molecular clock in SCN^{VIP} neurons. Related to Figure 2. Delta (δ: 0.5–5 Hz), theta (θ:5–9 Hz), sigma (σ: 9–15 Hz), beta (β: 15–30 Hz), low gamma (lγ: 30–60 Hz) and high gamma (hγ: 60–120 Hz) power bands (mean ± s.e.m) across 24 hrs of the 4th day in constant darkness in *VIP$^{cre/cre}$::Bmal1$^{fl/fl}$* (blue, n = 6) and

VIP$^{cret/wt}$*::Bmal1*$^{fl/fl}$ (red, n = 6) mice. Power bands were averaged for each 3 hr period and expressed as a percentage of the 24 hr average power for each frequency band and each vigilance state. * $p < 0.05$, ** $p < 0.01$, two-way ANOVA followed by a *post hoc* Bonferroni test.

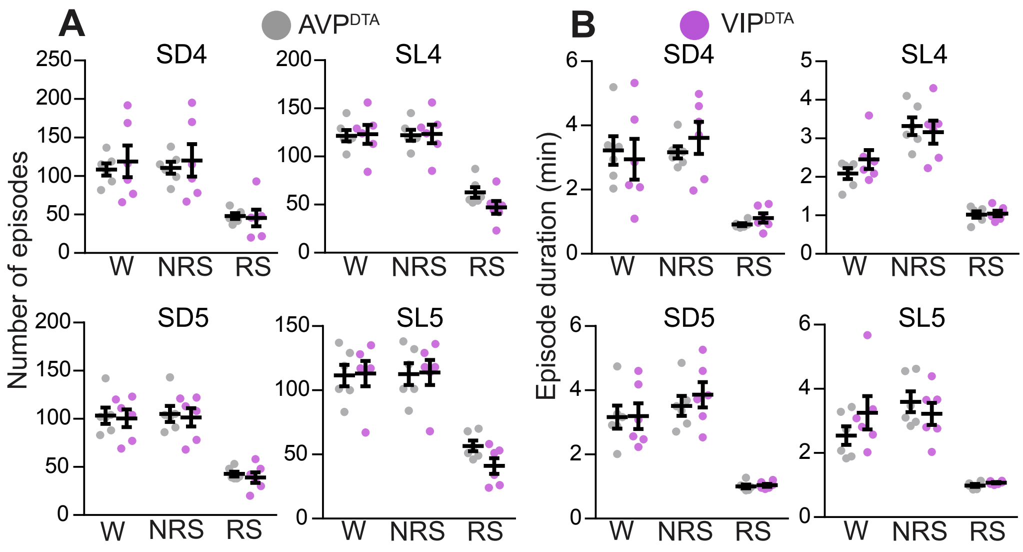

Figure S7. Sleep-wake qualitative changes following genetically-driven lesions of SCN^{VIP} or SCN^{AVP} neurons. Related to Figure 4. **(A)** Number of episodes (mean ± s.e.m.) of wake (W), NREM sleep (NRS) or REM sleep (RS) and **(B)** episode duration (mean ± s.e.m.) of W, NRS or RS during the 4th and 5th subjective dark (SD4, SD5), and 4th and 5th subjective light (SL4, SL5) periods in in AVP^{DTA} (grey, n = 6) and VIP^{DTA} (purple, n = 6) mice. $p > 0.05$, two-way ANOVA followed by a *post hoc* Bonferroni's test.

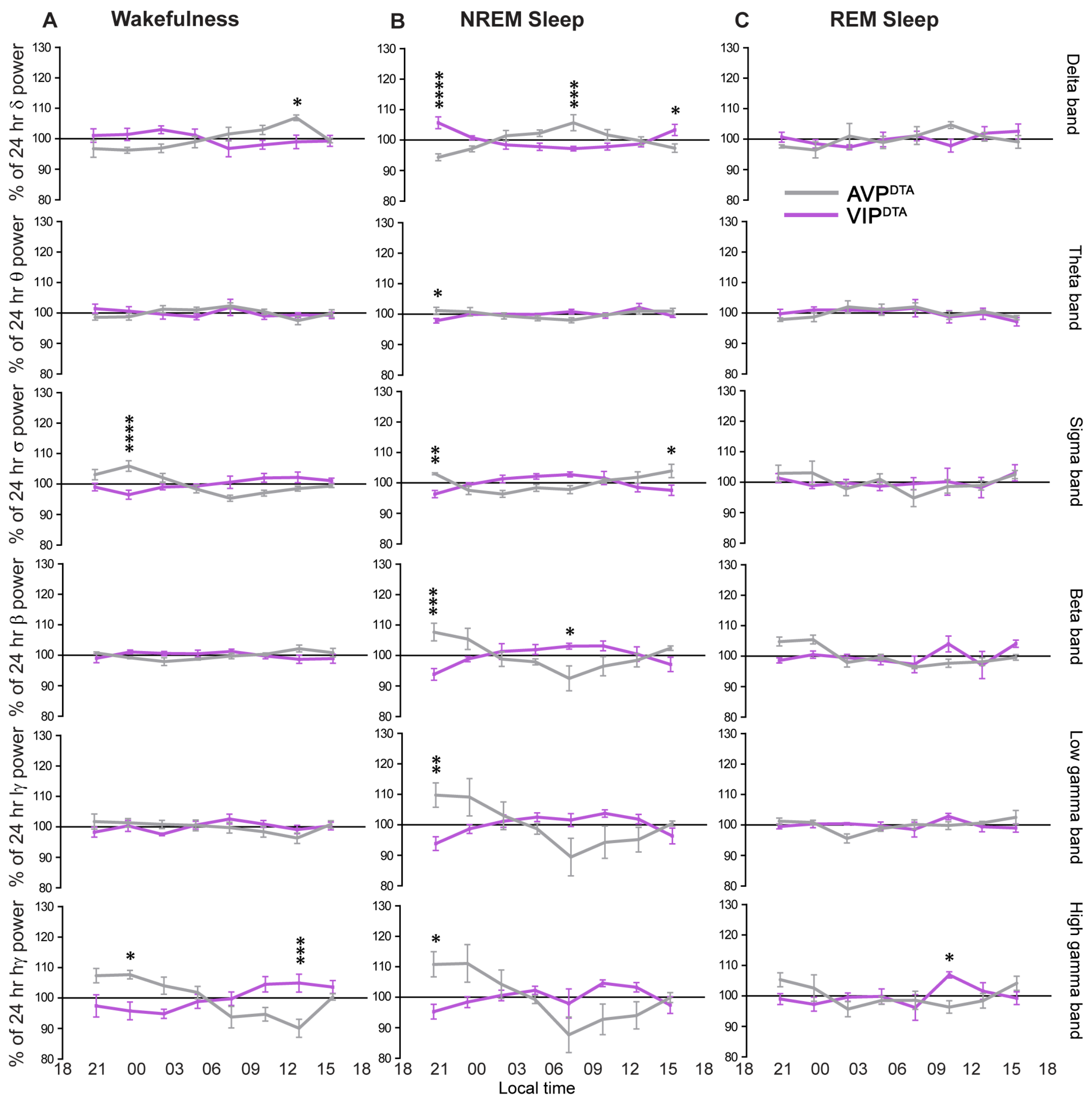

Figure S8. Sleep-wake cortical EEG power band activity across 24 hr period genetically-driven lesions of SCN^{VIP} or SCN^{AVP} neurons. Related to Figure 4. Delta (δ: 0.5–5 Hz), theta (θ:5–9 Hz), sigma (σ: 9–15 Hz), beta (β: 15–30 Hz), low gamma (lγ: 30–60 Hz) and high gamma (hγ: 60–120 Hz) power band (mean ± s.e.m.) activity across 24 hr of the 4th day in constant darkness in AVP^{DTA} (grey, n=5) and VIP^{DTA} (purple, n=6) mice. Power bands were averaged for each 3 hr period and expressed as a percentage

of the 24 hr average power for each frequency band and each vigilance state. * $p < 0.05$, ** $p < 0.01$, *** $p < 0.001$, **** $p < 0.0001$, two-way ANOVA followed by a *post hoc* Bonferroni test.

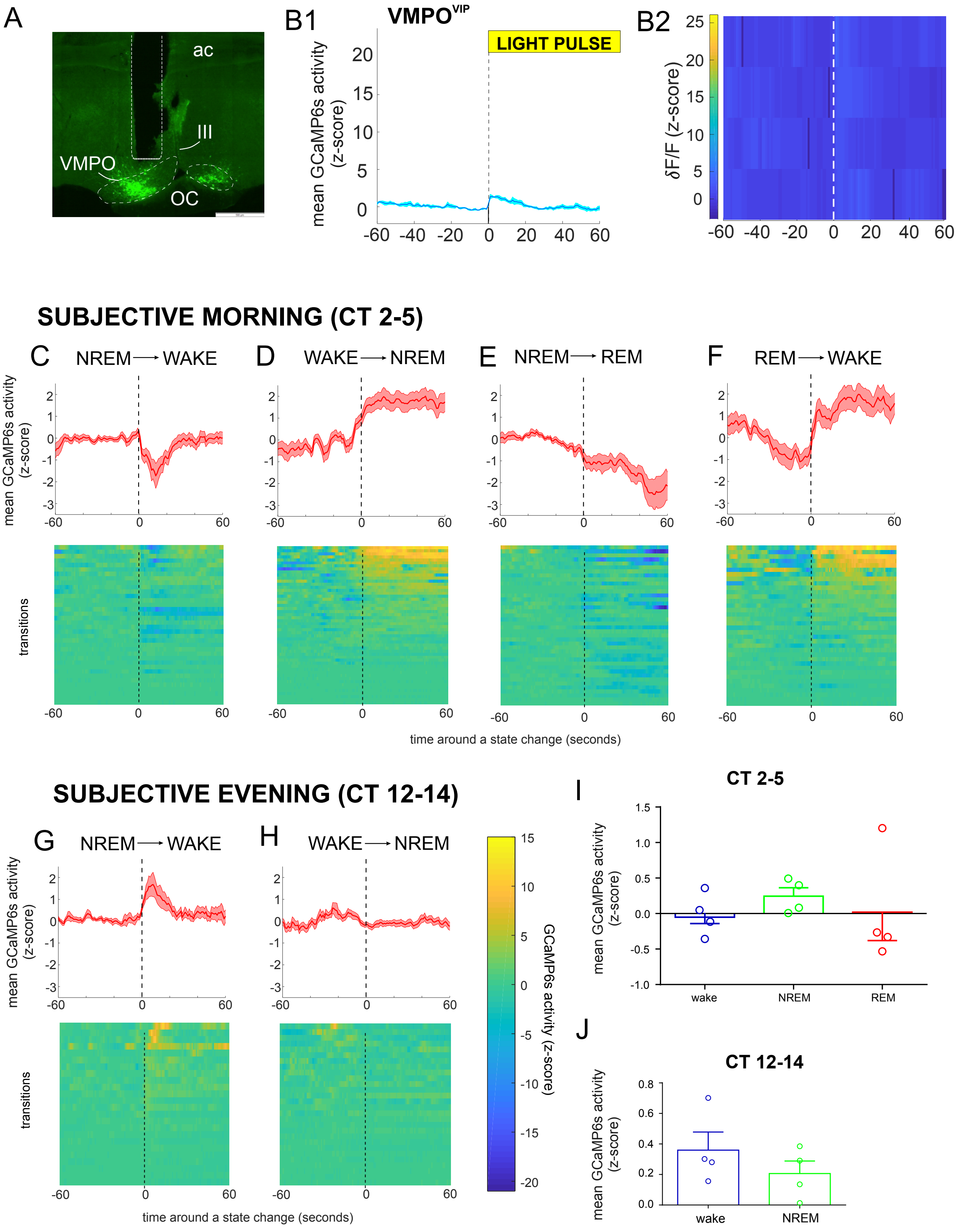
A
ac
III
VMPO
OC
B1
VMPO^VIP
LIGHT PULSE
mean GCaMP6s activity (z-score)
B2
δF/F (z-score)
SUBJECTIVE MORNING (CT 2-5)
C NREM → WAKE
D WAKE → NREM
E NREM → REM
F REM → WAKE
mean GCaMP6s activity (z-score)
transitions
time around a state change (seconds)
SUBJECTIVE EVENING (CT 12-14)
G NREM → WAKE
H WAKE → NREM
mean GCaMP6s activity (z-score)
transitions
time around a state change (seconds)
GCaMP6s activity (z-score)
I
CT 2-5
mean GCaMP6s activity (z-score)
wake
NREM
REM
J
CT 12-14
mean GCaMP6s activity (z-score)
wake
NREM

Figure S9. Activity in SCN^{VIP} neurons is behavioral state invariant. Related to Figure 7. (**A**) Photomicrograph depicting viral targeting to VIP-containing neurons in the ventromedial preoptic nucleus ($VMPO^{VIP}$) and placement of the photometry fiber. Abbreviations: III; 3rd ventricle, ac; anterior commissure, OC; optic chiasm (**B1**) Mean GCaMP6s activity in $VMPO^{VIP}$ neurons over all mice at the initiation of the light pulse Black dotted line indicates onset of the light pulse. Shaded area indicates s.e.m. **(B2)** Heatmap depicting GCaMP6s activity at the light pulse for each mouse. White dotted line indicates onset of the light pulse. (**C-J**) GCaMP6s activity at vigilance state transitions during the subjective morning (CT 2-5, C-F) and subjective evening (CT 12-14, G-H). Upper panels: means GCaMP6s activity across mice. Lower panels: Heatmaps depicting individual arousal state transitions from all mice. Paired t-tests (two-tailed) indicate no significant effect of the state transition upon GCaMP6s activity. (**I**) Mean (+ s.e.m) GCaMP6s activity during wake, NREM sleep and REM sleep between CT 2-5. RM one-way ANOVA indicated no significant effect of state upon GCaMP6s activity (Arousal state: $F(2, 6) = 0.2989$, $p = 0.7521$). (**J**) Mean (+ s.e.m) GCaMP6s activity during wake and NREM sleep between CT 12-14. Paired t-test (two-tailed) indicated no significant effect of state upon GCaMP6s activity ($p = 0.3128$, n = 4).

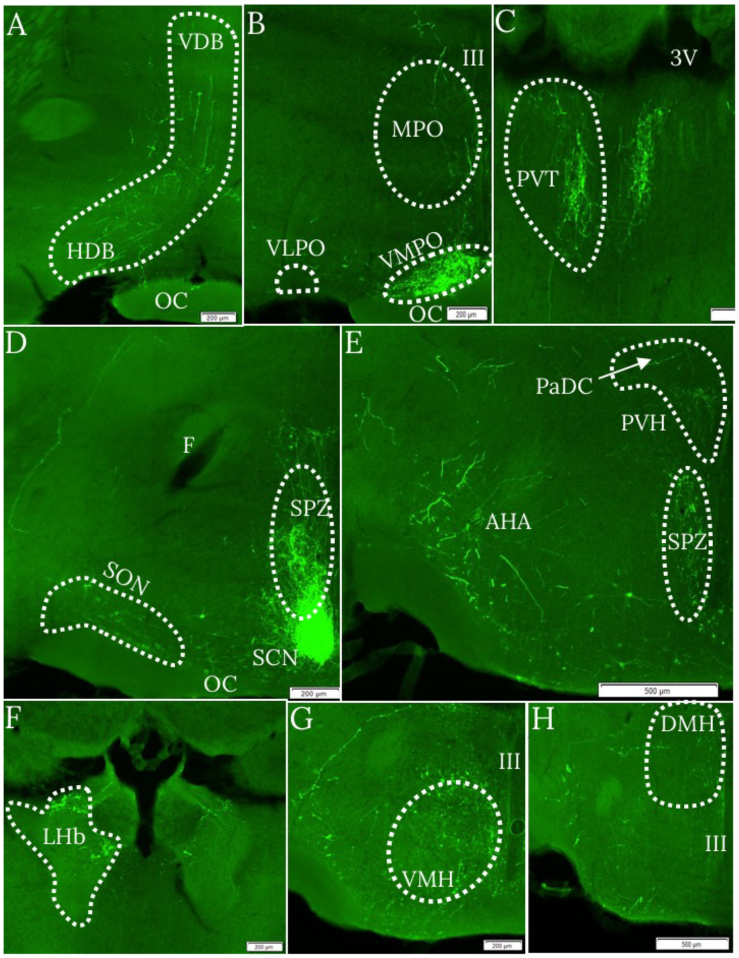

Brain Area	Terminal Density
Anterior Thalamus	+
Bed Nucleus of Stria Terminalis	+
Dorsomedial Hypothalamus	+++
Intergeniculate Leaflet of Thalamus	-
Lateroanterior Hypothalamic Nucleus	++++
Lateral Habenular Nucleus	++
Medial Habenular Nucleus	+
Medial Preoptic Area	++++
Median Preoptic Nucleus	+
Paraventricular Hypothalamus	++
Paraventricular Thalamus	+++
Lateral Hypothalamus	++
Periaqueductal Gray	+
Retromammillary Nucleus	++
Stria Terminalis	+++
Subparaventricular Zone	++++
Supraoptic Nucleus	++++
Ventrolateral Preoptic Nucleus	+
Ventromedial Hypothalamus	++++
Ventromedial Preoptic Nucleus	++++

Figure S10. SCN^{VIP} neurons project densely to the subparaventricular zone and provide innervation to many other hypothalamic and non-hypothalamic structures. Related to Figure 8. The anterograde tracer, AAV-FLEX-hrGFP was injected unilaterally into the SCN of *VIP-IRES-Cre* mice and axons filled with the tracer were assessed using a semi-quantitative method (see Methods) throughout the neuraxis. **(A-H)** Photomicrographs of hrGFP immunoreactivity (green) in the ventral diagonal band (VDB) and horizontal diagonal band (HDB) **(A)**, the ventromedial preoptic nucleus (VMPO) **(B)**, the paraventricular nucleus of the thalamus (PVT) **(C)**, the subparaventricular zone (SPZ) **(D)**, the paraventricular hypothalamus (PVH) and supraoptic nucleus (SON) **(E)**, the lateral habenula (LHb) **(F)**, the ventromedial hypothalamic nucleus (VMH) **(G)** and the

dorsomedial hypothalamic nucleus (DMH) **(H)**. **(I)** Table showing density of axons in each brain region. Abbreviations: III, 3rd ventricle; AHA, anterior hypothalamic area, anterior part; DMH, dorsomedial hypothalamic nucleus; HDB, nucleus of the horizontal limb of the diagonal band; LHb, lateral habenular nucleus; MPO, medial preoptic nucleus; OC, optic chiasm; PaDC, paraventricular hypothalamic nucleus, dorsal cap; PVH, paraventricular hypothalamic nucleus; PVT, paraventricular thalamic nucleus; SON, supraoptic nucleus; SPZ, subparaventricular zone of the hypothalamus; VDB, nucleus of the vertical limb of the diagonal band; VLPO, ventrolateral preoptic nucleus; VMH, ventromedial hypothalamic nucleus; VMPO, ventromedial preoptic nucleus.

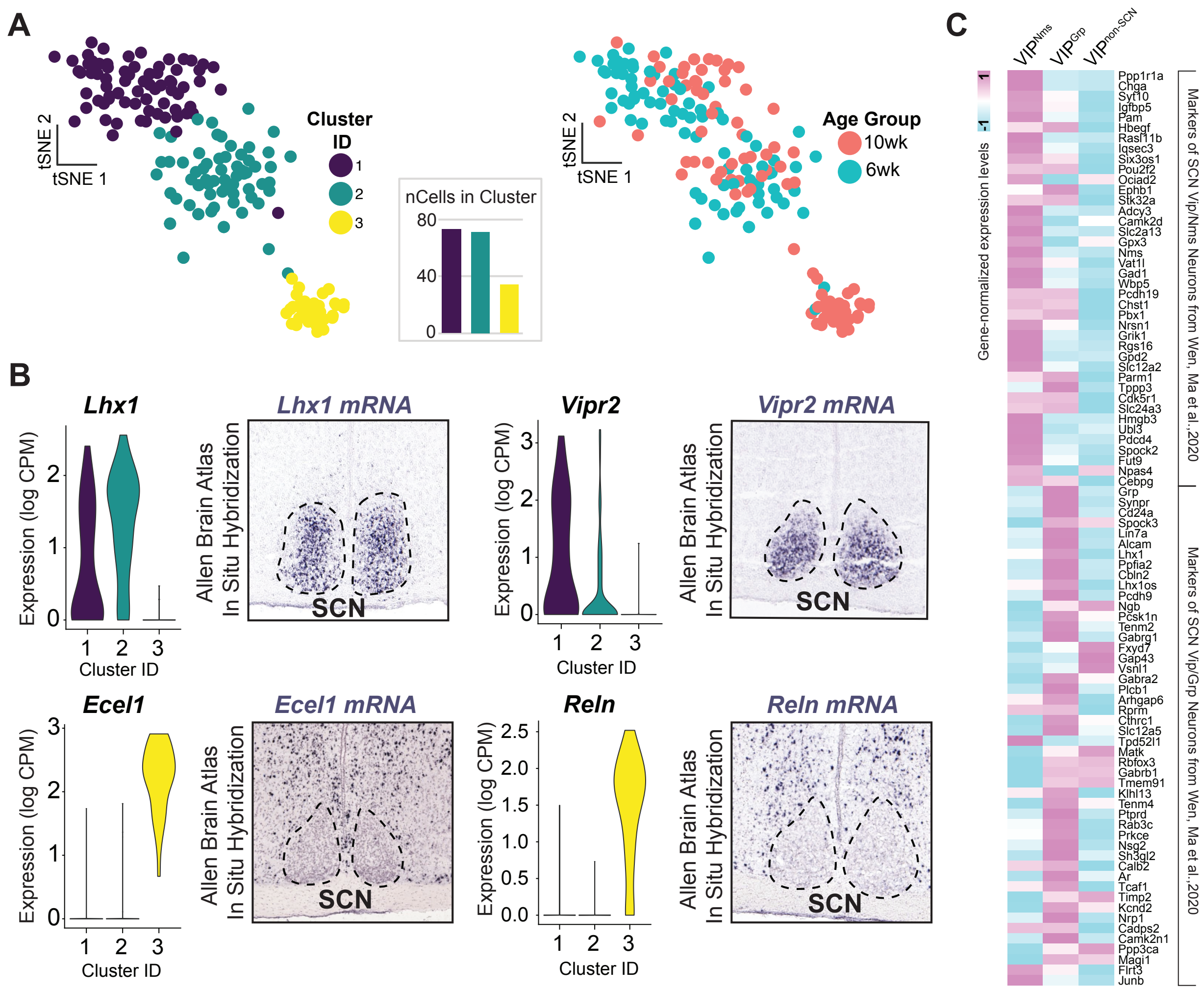

Figure S11. sNuc-seq analysis reveals three distinct VIP neuron populations, including two from the SCN. (A) t-Distributed Stochastic Neighbor Embedding (tSNE) plots of clustered VIP neurons, colored by cluster identity (left) or mouse age group (right). (B) Single-cell expression of genes enriched in or around the SCN, with corresponding in situ hybridization images from the Allen Mouse Brain Atlas: *Lhx1*, experiment 633; *Vipr2*, experiment 1104; *Ecel1*, experiment 70231305; and *Reln*, experiment 890. (C) Heatmap of cluster-level average expression (columns) of top marker genes (rows) for Vip/Grp neurons and Vip/Nms neurons identified by Wen, Ma et al., 2020[46].

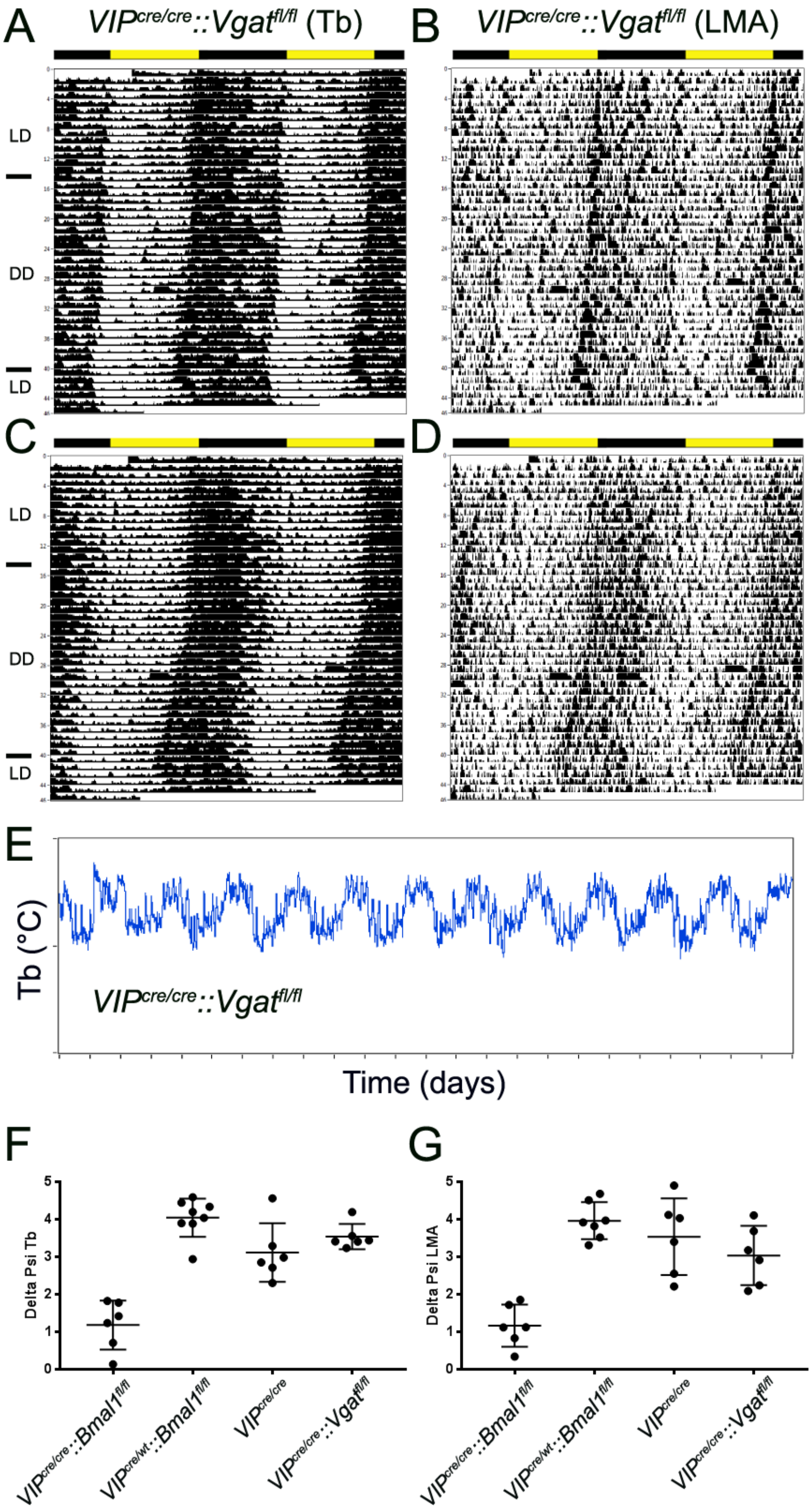

A
$VIP^{cre/cre}$::$Vgat^{fl/fl}$ (Tb)
B
$VIP^{cre/cre}$::$Vgat^{fl/fl}$ (LMA)
LD
DD
LD
C
D
E
Tb (°C)
$VIP^{cre/cre}$::$Vgat^{fl/fl}$
Time (days)
F
Delta Psi Tb
G
Delta Psi LMA
$VIP^{cre/cre}$::$Bmal1^{fl/fl}$
$VIP^{cre/wt}$::$Bmal1^{fl/fl}$
$VIP^{cre/cre}$
$VIP^{cre/cre}$::$Vgat^{fl/fl}$

Fig S12. Genetic disruption of GABAergic transmission from SCN^{VIP} neurons. (**A-D**) Selective deletion of *Vgat* in SCN^{VIP} neurons produced no discernable disruption in the diurnal (LD) or circadian (DD) rhythms of Tb or LMA (n=2 *VIP$^{cre/cre}$::Vgat$^{fl/fl}$* mice shown). (**E**) High amplitude circadian rhythms of Tb were observed in the *VIP$^{cre/cre}$::Vgat$^{fl/fl}$* (cf. with *VIP$^{cre/cre}$::Bmal1$^{fl/fl}$* mouse in Fig S3A). (**F-G**) deletion of *Vgat* in SCN^{VIP} neurons reduced the variability of the phase angle of entrainment of Tb, but not LMA, as compared to *VIP$^{cre/cre}$* mice (i.e., putative hypomorphic condition). *VIP^{crecre}::Bmal1$^{fl/fl}$,* n=6; *VIP$^{cre/wt}$::Bmal1$^{fl/fl}$,* n=7; *VIP$^{cre/cre}$*, n=6; *VIP$^{cre/cre}$::Vgat$^{fl/fl}$*, n=6. Means $\pm$ s.e.m.

Anatomical Abbreviations and Quantification for Figure 9

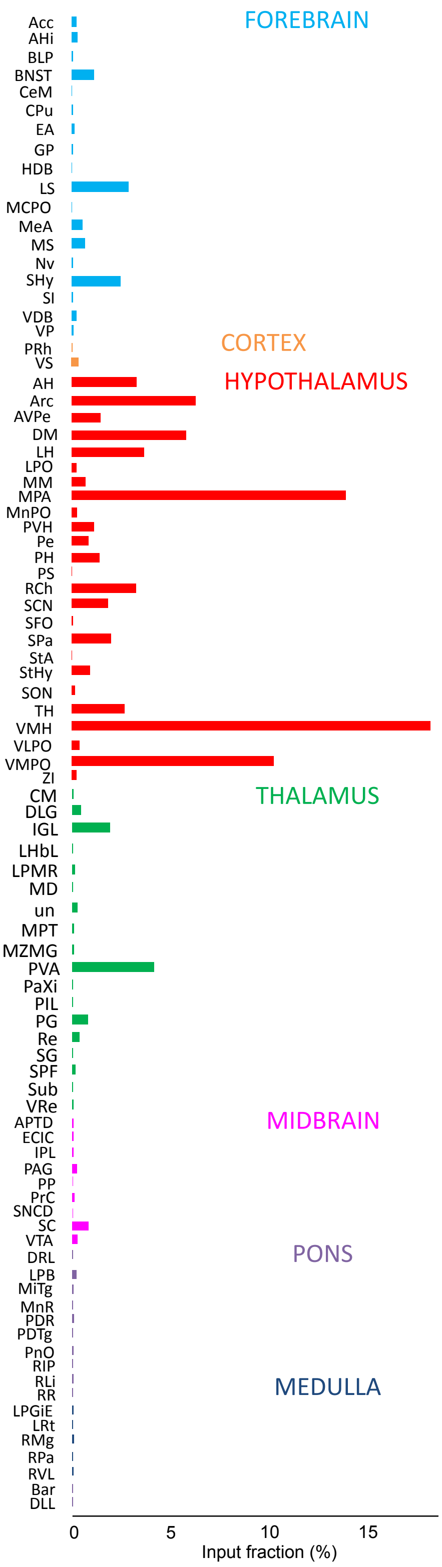

Abbreviation List: 3V, 3rd ventricle; ac, anterior commissure; Acc, accumbens nucleus; AH, anterior hypothalamic area, anterior part; Ahi, amygdalohippocampal area; alv, alveus of the hippocampus; APTD, anterior pretectal nucleus, dorsal part; Arc, arcuate hypothalamic nucleus; AVPe, anteroventral periventricular nucleus; Bar, Barrington's nucleus; BLP, basolateral amygdaloid nucleus, posterior part; BNST, bed nucleus of the stria terminalis; CeM, central amygdaloid nucleus, medial division; CM, central medial thalamic nucleus; CPu, caudate putamen (striatum); DLG, dorsal lateral geniculate nucleus; DLL, dorsal nucleus of the lateral lemniscus; DM, dorsomedial hypothalamic nucleus; DRL, dorsal raphe nucleus, lateral part; EA, extended amygdala; ECIC, external cortex of the inferior colliculus; fx, fornix; GP, globus pallidus; HDB, nucleus of the horizontal limb of the diagonal band; IGL, intergeniculate leaflet; IPL, interpeduncular nucleus, lateral subnucleus; LH, lateral hypothalamic area; LHbL, lateral habenular nucleus, lateral part; LPB, lateral parabrachial nucleus; LPGiE, lateral paragigantocellular nucleus, external part; LPMR, lateral posterior thalamic nucleus, mediorostral part; LPO, lateral preoptic area; LRt, lateral reticular nucleus; LSD, lateral septal nucleus, dorsal part; LSV, lateral septal nucleus, ventral part; LV, lateral ventricle; MCPO, magnocellular preoptic nucleus; MD, mediodorsal thalamic nucleus; MeA, medial amygdaloid nucleus, anterior part; MiTg, microcellular tegmental nucleus; MM, medial mammillary nucleus, medial part; MnPO, median preoptic nucleus; MnR, median raphe nucleus; MPA, medial preoptic area; MPT, medial pretectal nucleus; MS, medial septal nucleus; MZMG, marginal zone of the medial geniculate; Nv, navicular postolfactory nucleus; opt, optic tract; ox, optic chiasm; PAG, periaqueductal gray; PaXi, paraxiphoid nucleus of thalamus; PDR, posterodorsal raphe nucleus; PDTg,

posterodorsal tegmental nucleus; Pe, periventricular hypothalamic nucleus; PG, pregeniculate nucleus; PGMC, pregeniculate nucleus, magnocellular part; PGPC, pregeniculate nucleus, parvicellular part; PH, posterior hypothalamic nucleus; PIL, posterior intralaminar thalamic nucleus; PnO, pontine reticular nucleus, oral part; PP, peripeduncular nucleus; PrC, precommissural nucleus; PRh, perirhinal cortex; PS, parastrial nucleus; PVA, paraventricular thalamic nucleus, anterior part; PVH, paraventricular hypothalamic nucleus; PaDC, paraventricular hypothalamic nucleus, dorsal cap; Re, reuniens thalamic nucleus; RIP, raphe interpositus nucleus; RLi, rostral linear nucleus (midbrain); RMg, raphe magnus nucleus; RR, retrorubral nucleus; RVL, rostroventrolateral reticular nucleus; SC, superior colliculus; SCN, suprachiasmatic nucleus; SFO, subfornical organ; SG, suprageniculate thalamic nucleus; Shy, septohypothalamic nucleus; SHy, septohypothalamic nucleus; SI substantia innominate; SNCD, substantia nigra, compact part, dorsal tier; SON, supraoptic nucleus; Spa, subparaventricular zone of the hypothalamus; SPF, subparafascicular thalamic nucleus; StA, strial part of the preoptic area; STLI, bed nucleus of the stria terminalis, lateral division; STLP, bed nucleus of the stria terminalis, lateral division, posterior part; STLV, bed nucleus of the stria terminalis, lateral division, ventral part; STMA, bed nucleus of the stria terminalis, medial division, anterior part; STMAL, bed nucleus of the stria terminalis, medial division, anterolateral part; Sub, submedius thalamic nucleus; Te, terete hypothalamic nucleus; un, unassigned thalamic area, located between the fasciculus retroflexus; VDB, nucleus of the vertical limb of the diagonal band; VLPO, ventrolateral preoptic nucleus; VMH, ventromedial hypothalamic nucleus; VMPO, ventromedial preoptic nucleus; VP, ventral pallidum; VRe,ventral reuniens thalamic nucleus; VS, ventral subiculum; VTA, ventral tegmental area, ZI, zona incerta.
